# Supplementary material for: Comparison of outcomes between video laryngoscopy and flexible fiberoptic bronchoscopy for endotracheal intubation in adults with cervical neck immobilization: A systematic review and meta-analysis of randomized controlled trials
Source: PLoS One. 2024 Nov 15;19(11):e0313280. doi: 10.1371/journal.pone.0313280 (PMC11567517; doi:10.1371/journal.pone.0313280)
Supplement: S1 Checklist — (DOCX) [file pone.0313280.s001.docx]

| **Section and Topic** | **Item #** | **Checklist item** | **Location where item is reported** |
| --- | --- | --- | --- |
| **TITLE** | | |  |
| Title | 1 | Comparison of outcomes between video-laryngoscope and flexible fiberoptic bronchoscope for endotracheal intubation in adults with cervical neck immobilisation:A systematic review and meta-analysis of randomised controlled trials | 1 |
| **ABSTRACT** | | |  |
| Abstract | 2 | Purpose Comparing the outcomes of video-laryngoscopy and flexible fiberoptic bronchoscopy for endotracheal intubation in patients with cervical spine immobilization  Methods All of the comparative studies published in the PubMed, Cochrane Library, Medline, Web of Science, and EMBASE databases as of 8 Jan 2024 were included. All outcomes were analysed using Review Manager 5.4. The primary outcome was successful first-attempt intubation rate, intubation time, heart rate after intubation, mean arterial pressure after intubation, overall intubation success rate, risk of tissue damage and sore throat.  Results The meta-analysis included six randomized controlled studies with a total of 694 patients. The outcomes of the meta-analysis showed that the use of video-laryngoscopy was more better than flexible fiberoptic bronchoscope in terms of successful first-attempt intubation rate(P<0.05) and intubation time (P<0.05) in patients with cervical spine immobilization. However, there were no statistically significant differences in heart rate after intubation, mean arterial pressure after intubation, overall intubation success rate, risk of tissue damage, or sore throat (all P>0.05) between the video-laryngoscopy and flexible fiberoptic bronchoscopy groups. | 2 |
| **INTRODUCTION** | | |  |
| Rationale | 3 | Endotracheal intubation in patients with cervical spine instability requires special attention to prevent excessive motion of the cervical spine, which could result in secondary neurological damage [1-2]. Managing the airway in patients with suspected cervical instability poses a challenge for anesthesiologists. In trauma patients, the airway is typically secured through direct laryngoscopy. However, this procedure can cause movement of the cervical spine, potentially worsening existing injuries. To minimize the risk of spinal cord nerve damage during tracheal intubation, it is recommended to immobilize the cervical spine using a cervical collar [3-4]. However, cervical collar immobilization may increase the difficulty of direct laryngoscopic tracheal intubation because they limit the alignment of the three airway axes. Therefore, video laryngoscopy(VL) and flexible fiberoptic bronchoscopy (FFB) may be used to increase the success rate of tracheal intubation in patients with cervical spine immobilization [5]. Because they reduce cervical spine mobility in one step compared to direct laryngoscopy, they provide an additional safety measure in preventing aggravation of pre-existing injuries [6]. However, selecting the appropriate intubation device remains a clinical challenge due to the unique advantages and disadvantages associated with each device. For instance, using FFB often necessitates techniques to expand the retropharyngeal space, potentially resulting in varying degrees of cervical spine movement [7]. Moreover, utilizing FFB requires additional specialized training [8]. While VL exhibits less cervical extension compared to direct laryngoscopy, it may take longer to perform the intubation procedure.  1. Muckart DJ, Bhagwanjee S, van der Merwe R. Spinal cord injury as a result of endotracheal intubation in patients with undiagnosed cervical spine fractures. 1997;87(2):418-20.  2. Oppenlander ME, Hsu FD, Bolton P, Theodore N. Catastrophic neurological complications of emergent endotracheal intubation: report of 2 cases. J Neurosurg Spine. 2015;22(5):454-8.  3. Majernick TG, Bieniek R, Houston JB, Hughes HG. Cervical spine movement during orotracheal intubation. Ann Emerg Med. 1986;15(4):417-20.  4. Holmes MG, Dagal A, Feinstein BA, Joffe AM. Airway Management Practice in Adults With an Unstable Cervical Spine: The Harborview Medical Center Experience. Anesth Analg. 2018;127(2):450-454.  5. Rosenblatt WH, Wagner PJ, Ovassapian A, Kain ZN. Practice patterns in managing the difficult airway by anesthesiologists in the United States. Anesth Analg. 1998;87(1):153-7.  6. McElwain J, Laffey JG. Comparison of the C-MAC®, Airtraq®, and Macintosh laryngoscopes in patients undergoing tracheal intubation with cervical spine immobilization. Br J Anaesth. 2011;107(2):258-64.  7. Donaldson WF, Heil BV, Donaldson VP, Silvaggio VJ. The effect of airway maneuvers on the unstable C1-C2 segment. A cadaver study. Spine (Phila Pa 1976). 1997;22(11):1215-8.  8. Wong DM, Prabhu A, Chakraborty S, Tan G, Massicotte EM, Cooper R. Cervical spine motion during flexible bronchoscopy compared with the Lo-Pro GlideScope. Br J Anaesth. 2009;102(3):424-30.  9. Watts AD, Gelb AW, Bach DB, Pelz DM. Comparison of the Bullard and Macintosh laryngoscopes for endotracheal intubation of patients with a potential cervical spine injury. Anesthesiology. 1997;87(6):1335-42. | 3 |
| Objectives | 4 | Comparing the outcomes of video-laryngoscopy and flexible fiberoptic bronchoscopy for endotracheal intubation in patients with cervical spine immobilization | 3 |
| **METHODS** | | |  |
| Eligibility criteria | 5 | The study inclusion and exclusion processes were conducted in two groups. Initially, the selection was based on the title and abstract. If a decision could not be reached based on the summary, the full text of the article was retrieved. In cases where there was a disagreement between the two groups, the selection committee discussed the article until a consensus was reached. | 4 |
| Information sources | 6 | five electronic databases (PubMed, Cochrane Library, Medline, Web of Science, and EMBASE) | 4 |
| Search strategy | 7 | Systematic literature searches were conducted in five electronic databases (PubMed, Cochrane Library, Medline, Web of Science, and EMBASE) using a combination of MeSH (Medical Subject Heading) terms and free text words: “video laryngoscopy”, “flexible fiberoptic bronchoscopy” and “cervical collar”. The search was performed from the time databases were built until 8 Jan 2024, without any language or publication year restrictions. | 4 |
| Selection process | 8 | The study selection and exclusion process for the current meta-analysis is shown in Fig. 1 . We searched 642 studies from 5 electronic databases. Of these, 606 studies were excluded due to duplication (n=55) or irrelevance (n=551). After careful full-text evaluation, 6 studieswere reviewed, and the data were extracted. The demographic and clinical characteristics of the 6 studies are described in Table 1. A total of 348 patients underwent VL intubation, while 346 patients underwent FFB intubation. All patients used a cervical collar to fix the cervical spine. | 4 |
| Data collection process | 9 | Two reviewers used standardized data extraction tables. The extracted data included authors, publication date, title, country, study design, number of patients, mean age of patients, neck fixation technique, and comparison outcomes. The comparison outcomes included successful first-attempt intubation rate, intubation time, heart rate after intubation, mean arterial pressure after intubation, overall intubation success rate, risk of tissue damage, sore throat. All data were extracted from article texts, tables, and figures. The research author was contacted for missing data or further information. Two reviewers independently extracted the data; differences were resolved through discussion, and a consensus was reached by including third parties. | 4 |
| Data items | 10a | The comparison outcomes included successful first-attempt intubation rate, intubation time, heart rate after intubation, mean arterial pressure after intubation, overall intubation success rate, risk of tissue damage, sore throat. | 4 |
|  | 10b | All data were extracted from article texts, tables, and figures. The research author was contacted for missing data or further information. Two reviewers independently extracted the data; differences were resolved through discussion, and a consensus was reached by including third parties. | 5 |
| Study risk of bias assessment | 11 | For RCTs, we followed the guidelines outlined in the Cochrane Handbook for Systematic Reviews of Interventions, specifically focusing on 7 domains: random sequence generation, allocation concealment, blinding of participants and personnel, blinding of outcome assessment, incomplete outcome data, selective outcome reporting, and other sources of bias. The quality assessment was conducted by two reviewers independently, and any disagreements were resolved through discussion with a third party. | 6 |
| Effect measures | 12 | We used Review Manager Version 5.4 (Copenhagen: The Nordic Cochrane Centre, The Cochrane Collaboration) to analyse the data of all outcomes and compare the VL group with the FFB group. For continuous outcomes, such as intubation time, heart rate after intubation and mean arterial pressure after intubation, the means and standard deviations were pooled to a weighted mean difference (WMD) and 95% confidence interval (CI). Risk ratios (RRs) and 95% CIs were used to evaluate dichotomous outcomes, such as successful first-attempt intubation rate, overall intubation success rate, risk of tissue damage and sore throat. |  |
| Synthesis methods | 13a | Two reviewers used standardized data extraction tables. The extracted data included authors, publication date, title, country, study design, number of patients, mean age of patients, neck fixation technique, and comparison outcomes. The comparison outcomes included successful first-attempt intubation rate, intubation time, heart rate after intubation, mean arterial pressure after intubation, overall intubation success rate, risk of tissue damage, sore throat. All data were extracted from article texts, tables, and figures. The research author was contacted for missing data or further information. | 5 |
|  | 13b | The research author was contacted for missing data or further information. Two reviewers independently extracted the data; differences were resolved through discussion, and a consensus was reached by including third parties. | 5 |
|  | 13c | All data were extracted from article texts, tables, and figures. The research author was contacted for missing data or further information. | 5 |
|  | 13d | We used Review Manager Version 5.4 (Copenhagen: The Nordic Cochrane Centre, The Cochrane Collaboration) to analyse the data of all outcomes and compare the VL group with the FFB group. | 5 |
|  | 13e | We used I² to quantify heterogeneity. If I²>50%, the heterogeneity was significant, and the unstandardized mean difference was estimated using a random effects model. Otherwise, a fixed-effects model was applied. | 5 |
|  | 13f | removal of studies at high risk of bias, use of an alternative meta-analysis model | 5 |
| Reporting bias assessment | 14 | We used I² to quantify heterogeneity. If I²>50%, the heterogeneity was significant, and the unstandardized mean difference was estimated using a random effects model. Otherwise, a fixed-effects model was applied. | 5 |
| Certainty assessment | 15 | For continuous outcomes, such as intubation time, heart rate after intubation and mean arterial pressure after intubation, the means and standard deviations were pooled to a weighted mean difference (WMD) and 95% confidence interval (CI). Risk ratios (RRs) and 95% CIs were used to evaluate dichotomous outcomes, such as successful first-attempt intubation rate, overall intubation success rate, risk of tissue damage and sore throat. We used I² to quantify heterogeneity. | 6 |
| **RESULTS** | | |  |
| Study selection | 16a | The study selection and exclusion process for the current meta-analysis is shown in Fig. 1 . We searched 642 studies from 5 electronic databases. Of these, 606 studies were excluded due to duplication (n=55) or irrelevance (n=551). After careful full-text evaluation, 6 studies were reviewed, and the data were extracted. The demographic and clinical characteristics of the 6 studies are described in Table 1. A total of 348 patients underwent VL intubation, while 346 patients underwent FFB intubation. All patients used a cervical collar to fix the cervical spine. | 6 |
|  | 16b | We included studies that met the following criteria: (1). Included studies were RCTs.(2). A comparative study on the efficacy of VL and FFB in endotracheal intubation. (3). The comparison outcomes included at least one of the following: successful first-attempt intubation rate, intubation time, heart rate after intubation, mean arterial pressure after intubation, overall intubation success rate, risk of tissue damage, sore throat. | 6 |
| Study characteristics | 17 | Two reviewers used standardized data extraction tables. The extracted data included authors, publication date, title, country, study design, number of patients, mean age of patients, neck fixation technique, and comparison outcomes. The comparison outcomes included successful first-attempt intubation rate, intubation time, heart rate after intubation, mean arterial pressure after intubation, overall intubation success rate, risk of tissue damage, sore throat. All data were extracted from article texts, tables, and figures. The research author was contacted for missing data or further information. | 5 |
| Risk of bias in studies | 18 | Present assessments of risk of bias for each included study. | 6 |
| Results of individual studies | 19 | We used I² to quantify heterogeneity. If I²>50%, the heterogeneity was significant, and the unstandardized mean difference was estimated using a random effects model. Otherwise, a fixed-effects model was applied. | 5 |
| Results of syntheses | 20a | A total of 348 patients underwent VL intubation, while 346 patients underwent FFB intubation. All patients used a cervical collar to fix the cervical spine. Six studies reported intubation time, successful first-attempt intubation rate, and overall intubation success rate. Three studies reported mean arteria pressure after intubation. Heart rate after intubation was reported in 3 studies. Postintubation sore throat was reported in 4 studies. Risk of tissue damage were reported in 3 studies | 6 |
|  | 20b | Six studies with a total of 694 patients (VL group, n=348 vs. FFB group, n=346) compared the mean intubation time. The meta-analysis indicated that the VL group had significantly fewer intubation times than the FFB group (WMD, -27.07; 95% CI, -34.01 to -20.13; P<0.05). The heterogeneity test outcome (I²=94%) indicated significant heterogeneity. | 6 |
|  | 20c | If investigations of possible causes of heterogeneity were conducted: o present results regardless of the statistical significance, magnitude, or direction of effect modification. o identify the studies contributing to each subgroup. o report results with due consideration to the observational nature of the analysis and risk of confounding due to other factors | 6 |
|  | 20d | We used I² to quantify heterogeneity. If I²>50%, the heterogeneity was significant, and the unstandardized mean difference was estimated using a random effects model. Otherwise, a fixed-effects model was applied. | 5 |
| Reporting biases | 21 | sensitivity analyses seeking to explore the potential impact of missing results on the synthesis were conducted, present results of each analysis, compare them with results of the primary analysis, and report results with due consideration of the limitations of the statistical method. | 6 |
| Certainty of evidence | 22 | The study selection and exclusion process for the current meta-analysis is shown in Fig. 1 . We searched 642 studies from 5 electronic databases. Of these, 606 studies were excluded due to duplication (n=55) or irrelevance (n=551). | 6 |
| **DISCUSSION** | | |  |
| Discussion | 23 | One limitation of this meta-analysis is that the studies included did not involve actual trauma patients and did not utilize rapid sequence induction procedures. As a result, the findings of this study may not be applicable to all patients with cervical spine immobilization in real-life situations. Furthermore, it is possible that the unfamiliarity of clinicians with FFB intubation techniques, in comparison to VL, could have introduced bias into our findings. | 8 |
| **OTHER INFORMATION** | | |  |
| Registration and protocol | 24 | Systematic review protocol：CRD42024499868 | 3 |
| Support | 25 | None |  |
| Competing interests | 26 | The authors declare no conflict exists | 23 |
| Availability of data, code and other materials | 27 | The datasets used and/or analysed during the current study are available from the corresponding author on reasonable reque | 23 |

*From:*  Page MJ, McKenzie JE, Bossuyt PM, Boutron I, Hoffmann TC, Mulrow CD, et al. The PRISMA 2020 statement: an updated guideline for reporting systematic reviews. BMJ 2021;372:n71. doi: 10.1136/bmj.n71
