# Supplementary material for: Comparison of outcomes between video laryngoscopy and flexible fiberoptic bronchoscopy for endotracheal intubation in adults with cervical neck immobilization: A systematic review and meta-analysis of randomized controlled trials
Source: PLoS One. 2024 Nov 15;19(11):e0313280. doi: 10.1371/journal.pone.0313280 (PMC11567517; doi:10.1371/journal.pone.0313280)
Supplement: S1 Table — (DOCX) [file pone.0313280.s002.docx]

**S4 Table.** List of Included and Excluded Studies.

| Included and excluded reasons | No. studies | References. |
| --- | --- | --- |
| Included studies | 6 | [1-6] |
| Duplicate studies | 55 | [7-61] |
| Irrelevant studies | 551 | [62-612] |
| Not meet the inclusion criteria | 21 | [622-633 |
| In vitro study | 5 | [634-638 |
| Can't extract data | 4 | [639-642] |

REFERENCES

1.Abdullah HR, Li-Ming T, Marriott A, Wong TG. A comparison between the Bonfils Intubation Fiberscope and McCoy laryngoscope for tracheal intubation in patients with a simulated difficult airway. Anesth Analg. 2013;117(5):1217-20.

2.Yumul R, Elvir-Lazo OL, White PF, Durra O, Ternian A, Tamman R, et al. Comparison of the C-MAC video laryngoscope to a flexible fiberoptic scope for intubation with cervical spine immobilization. J Clin Anesth. 2016;31:46-52.

3. Tung A, Griesdale DE. Comparing the novel GlideScope Groove videolaryngoscope with conventional videolaryngoscopy: a randomized mannequin study of novice providers. J Clin Anesth. 2013;25(8):644-50.

4.. Shulman GB, Connelly NR. A comparison of the Bullard laryngoscope versus the flexible fiberoptic bronchoscope during intubation in patients afforded inline stabilization. J Clin Anesth. 2001;13(3):182-5.

5.. Choi S, Yoo HK, Shin KW, Kim YJ, Yoon HK, Park HP, et al. Videolaryngoscopy vs. flexible fibrescopy for tracheal intubation in patients with cervical spine immobilisation: a randomised controlled trial. Anaesthesia. 2023;78(8):970-978.

6.. Gill N, Purohit S, Kalra P, Lall T, Khare A. Comparison of hemodynamic responses to intubation: Flexible fiberoptic bronchoscope versus McCoy laryngoscope in presence of rigid cervical collar simulating cervical immobilization for traumatic cervical spine. Anesth Essays Res. 2015;9(3):337-42.

7.(2012). "7th EuroNeuro Congress." EUROPEAN JOURNAL OF ANAESTHESIOLOGY 29.

8.(2013). "16th Annual Society for Airway Management Scientific Meeting, SAM 2012." JOURNAL OF CLINICAL ANESTHESIA 25(3).

9.(2013). "Annual Meeting of the Scottish Airway Group 2013." Anaesthesia 68(8).

10Abdallah, C., et al. (2010). "Laryngeal fracture in a pediatric patient." ANESTHESIA AND ANALGESIA 110(3): S120.

11.Abdellatif, A. A. and M. A. Ali (2014). "GlideScope videolaryngoscope versus flexible fiberoptic bronchoscope for awake intubation of morbidly obese patient with predicted difficult intubation." Middle East journal of anaesthesiology 22(4): 385‐392.

12.Aghdaii, N., et al. (2010). "Cardiovascular responses to orotracheal intubation in patients undergoing coronary artery bypass grafting surgery. Comparing fiberoptic bronchoscopy with direct laryngoscopy." Middle East journal of anaesthesiology 20(6): 833‐838.

13.Agrawal, N., et al. (2021). "Comparison of C-MAC D-Blade with macintosh laryngoscope for endotracheal intubation in patients with cervical spine immobilization: A randomized controlled trial." Trends in Anaesthesia and Critical Care 37: 35-41

14.Agrawal, S., et al. (2018). "O-C1-C2 dynamics during flexible fiberoptic bronchoscopy and video laryngoscopy in patients with atlantoaxial dislocation: A cinefluroscopic comparison." JOURNAL OF NEUROSURGICAL ANESTHESIOLOGY 30(4): 416.

15.Agrawal, S., et al. (2019). "O-C1-C2 dynamics during flexible fiberoptic bronchoscopy and video laryngoscopy in patients with cranio-vertebral junction pathology (Atlanto-axial dislocation/Basilar invagination): a cinefluroscopic comparison." JOURNAL OF NEUROSURGICAL ANESTHESIOLOGY 31(4): 507.

16.Agrawal, S., et al. (2021). "Fiberoptic bronchoscopy versus video laryngoscopy guided intubation in patients with craniovertebral junction instability: A cinefluroscopic comparison." Surg Neurol Int 12: 92.

17.Agrawal, S., et al. (2021). "Fiberoptic bronchoscopy versus video laryngoscopy guided intubation in patients with craniovertebral junction instability: a cinefluroscopic comparison." Surgical Neurology International 12.

18.Agrò, F., et al. (2003). "Tracheal intubation using a Macintosh laryngoscope or a GlideScope in 15 patients with cervical spine immobilization." Br J Anaesth 90(5): 705-706.

19.Agrò, F., et al. (2001). "The esophageal tracheal combitube as a non-invasive alternative to endotracheal intubation. A review." Minerva Anestesiol 67(12): 863-874.

20.Ahmad, N., et al. (2005). "Management of difficult intubation in a patient with ankylosing spondylitis: A case report." Middle East Journal of Anesthesiology 18(2): 379-384.

21.Ahmad, S., et al. (2022). "Awake tracheal intubation using king Vision video laryngoscope in traumatic cervical spine patients: A case series." Trends in Anaesthesia and Critical Care 45: 55-57.

22.Akbar, S. H. and J. S. Ooi (2015). "COMPARISON BETWEEN C-MAC VIDEO-LARYNGOSCOPE AND MACINTOSH DIRECT LARYNGOSCOPE DURING CERVICAL SPINE IMMOBILIZATION." Middle East J Anaesthesiol 23(1): 43-50.

23.Akhaddar, A., et al. (2020). "Unilateral blindness following superior laryngeal nerve block for awake tracheal intubation in a case of posterior cervical spine surgery." Surg Neurol Int 11: 277.

24.Alam, A., et al. (2022). "Fungal Empyema Thoracis in an Immunocompetent Patient with Suspected Drug Induced Pneumonitis." American Journal of Respiratory and Critical Care Medicine 205(1).

25.Albalbissi, A. A., et al. (2019). "Bronchoscopy leading to coma and quadriparesis!! the secret revealed." American Journal of Respiratory and Critical Care Medicine 199(9).

26.Aleksandrowicz, D. and T. Gaszynski (2016). "Airway Management with Cervical Spine Immobilisation: A Comparison between the Macintosh Laryngoscope, Truview Evo2, and Totaltrack VLM Used by Novices-A Manikin Study." BioMed research international 2016.

27.Aleksandrowicz, D. and T. Gaszyński (2016). "Airway Management with Cervical Spine Immobilisation: A Comparison between the Macintosh Laryngoscope, Truview Evo2, and Totaltrack VLM Used by Novices--A Manikin Study." Biomed Res Int 2016: 1297527.

28.Aleksandrowicz, D. and T. Gaszyński (2017). "Airway management with simultaneous cervical spine immobilisation: A comparison between the Macintosh laryngoscope and the Airtraq® optical laryngoscope used by experienced paramedics – A manikin study." Trends in Anaesthesia and Critical Care 15: 25-28.

29.Aleksandrowicz, D. and T. Gaszyński (2018). "The pressure exerted on the tongue during intubation with simultaneous cervical spine immobilisation: a comparison between four videolaryngoscopes and the Macintosh laryngoscope-a manikin study." J Clin Monit Comput 32(5): 907-913.

30.Aleksandrowicz, D. and T. Gaszyński (2018). "The pressure exerted on the tongue during intubation with simultaneous cervical spine immobilisation: a comparison between four videolaryngoscopes and the Macintosh laryngoscope—a manikin study." JOURNAL OF CLINICAL MONITORING AND COMPUTING 32(5): 907-913.

31.Aleksandrowicz, D. and T. Gaszyński (2018). "Tracheal intubation in a simulated cervical spine immobilisation: The Macintosh laryngoscope versus supraglottic airway devices - A manikin study." Trends in Anaesthesia and Critical Care 21: 53-56

32.Aleksandrowicz, D., et al. (2018). "Intubation with cervical spine immobilisation: a comparison between the KingVision videolaryngoscope and the Macintosh laryngoscope: A randomised controlled trial." Eur J Anaesthesiol 35(5): 399-401.

33.Ali, Q. E., et al. (2017). "A comparative evaluation of King Vision video laryngoscope (Channelled blade), McCoy, and Macintosh laryngoscopes for tracheal intubation in patients with immobilized cervical spine." Sri Lankan Journal of Anaesthesiology 25(2): 70-75.

34.Ali, Q. E., et al. (2017). "King vision video laryngoscope: A suitable device for severe ankylosing spondylitis." Egyptian journal of anaesthesia 33(1): 129-131.

35.Amathieu, R., et al. (2012). "Simulating face-to-face tracheal intubation of a trapped patient: a randomized comparison of the LMA Fastrach&trade;, the GlideScope&trade;, and the Airtraq&trade; laryngoscope." Br J Anaesth 108(1): 140-145.

36.Amathieu, R., et al. (2012). "Simulating face-to-face tracheal intubation of a trapped patient: a randomized comparison of the LMA Fastrach™, the GlideScope™, and the Airtraq™ laryngoscope." BRITISH JOURNAL OF ANAESTHESIA 108(1): 140-145.

37.Ambrosio, A., et al. (2014). "Difficult airway management for novice physicians: a randomized trial comparing direct and video-assisted laryngoscopy." Otolaryngol Head Neck Surg 150(5): 775-778.

38.Ambrosio, A. A., et al. (2013). "Difficult airway simulation for novice physicians: A randomized trial comparing traditional and video-assisted laryngoscopy." Otolaryngology - Head and Neck Surgery (United States) 149(2): P150.

39.Amor, M., et al. (2013). "A comparison of Airtraq™ laryngoscope and standard direct laryngoscopy in adult patients with immobilized cervical spine." Annales francaises d'anesthesie et de reanimation 32(5): 296-301.

40.Aoi, Y., et al. (2010). "Airway scope versus macintosh laryngoscope in patients with simulated limitation of neck movements." Journal of Trauma - Injury, Infection and Critical Care 69(4): 838-842.

41.Aoi, Y., et al. (2010). "Airway scope laryngoscopy under manual in-line stabilization and cervical collar immobilization: A cross-over in vivo cinefluoroscopic study." ANESTHESIA AND ANALGESIA 110(3): S104.

42.Appachi, S., et al. (2022). "An Analysis of Tracheostomy Complications in Pediatric Patients With Scoliosis." LARYNGOSCOPE 132(5): 944-948.

43.Arora, D., et al. (2006). "Cervical spine hyperostosis: An unusual cause of difficult intubation during coronary artery bypass grafting." Journal of Anaesthesiology Clinical Pharmacology 22(4): 411-414.

44.Arumugam, S. K., et al. (2011). "Malignant hyperpyrexia in cervical spine injury." Qatar Medical Journal 20(2): 62-63.

45.Averill, L., et al. (2016). "Imaging of the thoracic inlet in morquio a syndrome." Pediatric radiology 46: S211.

46.Avitsian, R., et al. (2006). "Successful reintubation after cervical spine exposure using an Aintree intubation catheter and a Laryngeal Mask Airway." JOURNAL OF CLINICAL ANESTHESIA 18(3): 224-225.

47.Aydin, K., et al. (2012). "Cervical screw extrusion into the trachea leading to perforation: A case report." Chirurgia (Turin) 25(5): 355-357.

48.Bacon, E. R., et al. (2015). "Tips and Troubleshooting for Use of the GlideScope Video Laryngoscope for Emergency Endotracheal Intubation." Am J Emerg Med 33(9): 1273-1277.

49.Bair, A. E., et al. (2010). "Assessment of the storz video Macintosh laryngoscope for use in difficult airways: A human simulator study." Acad Emerg Med 17(10): 1134-1137.

50.Bakshi, S. G., et al. (2019). "McGrath MAC video laryngoscope versus direct laryngoscopy for the placement of double-lumen tubes: a randomised control trial." Indian Journal of Anaesthesia 63(6): 456‐461.

51.Bala, R., et al. (2023). "Comparison between Air Q and intubating laryngeal mask airway as intubation conduits in patients with simulated fixed cervical spine: a prospective observational study." Medical Gas Research 13(1): 10-14.

52.Balk, R. A. (1997). "The technique of orotracheal intubation: Twenty seconds that can save a patient's life." Journal of Critical Illness 12(5): 316-323.

53.Bamshad, M., et al. (1989). "Acute upper airway obstruction in rheumatoid arthritis of the cricoarytenoid joints." South Med J 82(4): 507-511.

54.Bamshad, M., et al. (1989). "Acute upper airway obstruction in rheumatoid arthritis of the cricoarytenoid joints." SOUTHERN MEDICAL JOURNAL 82(4): 507-511.

55.Barch, B., et al. (2012). "Difficult pediatric airway management using the intubating laryngeal airway." Int J Pediatr Otorhinolaryngol 76(11): 1579-1582.

56.Barch, B. E., et al. (2011). "A new algorithm for pediatric airway management using the intubating laryngeal airway." Otolaryngology - Head and Neck Surgery 145: 105.

57.Bartolek, D., et al. (2009). "Life threatening complications after unsuccessful attempt of the guidewire dilating forceps tracheostomy in multi-trauma patient with cervical spine injury." Coll Antropol 33(4): 1409-1413.

58.Bartolek, D., et al. (2009). "Life Threatening Complications after Unsuccessful Attempt of the Guidewire Dilating Forceps Tracheostomy in Multi-Trauma Patient with Cervical Spine Injury." COLLEGIUM ANTROPOLOGICUM 33(4): 1409-1413.

59.Basra, S. K., et al. (2006). "Methemoglobinemia after fiberoptic intubation in a patient with an unstable cervical fracture - A case report." JOURNAL OF SPINAL DISORDERS & TECHNIQUES 19(4): 302-304.

60.Basra, S. K., et al. (2006). "Methemoglobinemia after fiberoptic intubation in a patient with an unstable cervical fracture: a case report." J Spinal Disord Tech 19(4): 302-304.

61.Bathory, I., et al. (2009). "Evaluation of the GlideScope for tracheal intubation in patients with cervical spine immobilisation by a semi-rigid collar." Anaesthesia 64(12): 1337-1341.

62.Bathory, I., et al. (2009). "Evaluation of the GlideScope® for tracheal intubation in patients with cervical spine immobilisation by a semi-rigid collar." Anaesthesia 64(12): 1337-1341.

63.Bathory, I., et al. (2009). "Evaluation of the GlideScope® for tracheal intubation in patients with cervical spine immobilization by a semi-rigid collar." EUROPEAN JOURNAL OF ANAESTHESIOLOGY 26: 222-223.

64.Becker, S. A. and I. Maldonado (2010). "Safety of percutaneous tracheostomy in trauma patients." Intensive care medicine 36: S299.

65.Bharadwaj, A., et al. (2016). "Cervical Spine Movement and Ease of Intubation Using Truview or McCoy Laryngoscope in Difficult Intubation." Spine (Phila Pa 1976) 41(12): 987-993.

66.Bhardwaj, N., et al. (2013). "Assessment of cervical spine movement during laryngoscopy with Macintosh and Truview laryngoscopes." J Anaesthesiol Clin Pharmacol 29(3): 308-312.

67.Bhardwaj, Y. and A. Singam (2023). "Comparative Preference of Airtraq Laryngoscope Over Macintosh Laryngoscope- A Review." Journal of Clinical and Diagnostic Research 17(1): UE01-UE04.

68.Bharti, N. and S. Arora (2012). "Airway management in patients with immobilized cervical spine: A comparison of macintosh, maccoy and truview laryngoscope." EUROPEAN JOURNAL OF ANAESTHESIOLOGY 29: S1.

69.Bharti, N., et al. (2014). "A comparison of McCoy, TruView, and Macintosh laryngoscopes for tracheal intubation in patients with immobilized cervical spine." Saudi J Anaesth 8(2): 188-192.

70.Bhatnagar, S., et al. (2005). "The LMA Fastrach facilitates fibreoptic intubation in oral cancer patients." Journal canadien d'anesthesie [Canadian journal of anaesthesia] 52(6): 641‐645.

71.Bhola, R., et al. (2014). "Tracheal intubation in patients with cervical spine immobilization: A comparison of McGrath(®) video laryngoscope and Truview EVO2(®) laryngoscope." Indian J Anaesth 58(3): 269-274.

72.Bilbao Ares, A., et al. (2017). "Airway management in patients with cervical spine trauma and neurological symptoms. Case reports." Revista Colombiana de Anestesiologia 45: 45-49.

73.Bilgin, H. and M. Bozkurt (2006). "Tracheal intubation using the ILMA, C-Trach or McCoy laryngoscope in patients with simulated cervical spine injury." Anaesthesia 61(7): 685-691.

74.Bilgin, H. and M. Bozkurt (2006). "Tracheal intubation using the ILMA, C-Trach™ or McCoy laryngoscope in patients with simulated cervical spine injury." Anaesthesia 61(7): 685-691.

Bjoernsen, L. P. and B. Lindsay (2009). "Video laryngoscopy in the prehospital setting." Prehosp Disaster Med 24(3): 265-270.

75.Bogdanski, L., et al. (2015). "Simulated endotracheal intubation of a patient with cervical spine immobilization during resuscitation: a randomized comparison of the Pentax AWS, the Airtraq, and the McCoy Laryngoscopes." AMERICAN JOURNAL OF EMERGENCY MEDICINE 33(12): 1814-1817.

76.Bois, M. C., et al. (2014). "Increasing Pulmonary Infiltrates in a 72-Year-Old Woman With Metastatic Breast Cancer." Chest 146(6): e208-e211.

77.Borland, L. M. (1988). "Establishing the pediatric airway." International Anesthesiology Clinics 26(1): 27-31.

78.Brimacombe, J. and A. Berry (1993). "Laryngeal mask airway insertion. A comparison of the standard versus neutral position in normal patients with a view to its use in cervical spine instability." Anaesthesia 48(8): 670-671.

79.Brimacombe, J., et al. (2000). "Cervical spine motion during airway management: a cinefluoroscopic study of the posteriorly destabilized third cervical vertebrae in human cadavers." Anesth Analg 91(5): 1274-1278.

80.Byhahn, C., et al. (2010). "Tracheal intubation using the mobile C-MAC video laryngoscope or direct laryngoscopy for patients with a simulated difficult airway." Minerva Anestesiol 76(8): 577-583.

81.Byhahn, C., et al. (2008). "Brief report: Tracheal intubation using the Bonfils intubation fibrescope or direct laryngoscopy for patients with a simulated difficult airway." Canadian Journal of Anesthesia 55(4): 232-237.

82.Byhahn, C., et al. (2008). "Brief report: Tracheal intubation using the Bonfils intubation fibrescope or direct laryngoscopy for patients with a simulated difficult airway." CANADIAN JOURNAL OF ANAESTHESIA-JOURNAL CANADIEN D ANESTHESIE 55(4): 232-237.

83.Campbell-Lloyd, A. J. M., et al. (2009). "Vertebral artery dissection after direct laryngoscopy: Case report and literature review." Journal of laryngology and otology 123(2).

84.Carley, S. and J. Butler (2000). "Towards evidence based emergency medicine:Best BETs from the Manchester Royal Infirmary. Use of the McCoy laryngoscope in patients with suspected cervical spine fracture." J Accid Emerg Med 17(5): 364-365.

85.Caruso, T., et al. (2018). "The incidence of desaturation during microlaryngoscopy and bronchoscopy: a quality control review." ANESTHESIA AND ANALGESIA 126(4): 572.

86.Cetin, Y. S. and C. Soyalp (2022). "Comparison of three video-assisted intubation methods: rigid telescopes, C-MAC, flexible fiberoptic bronchoscopy, for anticipated difficult airways." Trends in Anaesthesia and Critical Care 43: 33‐37.

87.Chalam, K. and J. Gupta (2016). "Comparison of intubating laryngeal mask airway and fiberoptic bronchoscopy for endotracheal intubation in patients undergoing cervical discectomy." Journal of anaesthesiology, clinical pharmacology 32(4): 515‐518.

88.Chalam, K. S. and J. Gupta (2016). "Comparison of intubating laryngeal mask airway and fiberoptic bronchoscopy for endotracheal intubation in patients undergoing cervical discectomy." J Anaesthesiol Clin Pharmacol 32(4): 515-518.

89.Chandrashekaraiah, M. M., et al. (2021). "Simulated difficult airway: Cmac d blade or glidescope?" Sri Lankan Journal of Anaesthesiology 29(1): 7-12.

90.Chandrashekaraiah, M. M., et al. (2017). "Evaluation of ease of intubation using C-MAC vs Macintosh laryngoscope in patients with the application of manual inline axial stabilization – A randomized comparative study." Sri Lankan Journal of Anaesthesiology 25(1): 8-12.

91.Chandy, J., et al. (2021). "A randomized clinical trial comparing the King Vision (channeled blade) and the CMAC (D blade) videolaryngoscopes in patients with cervical spine immobilization." J Anaesthesiol Clin Pharmacol 37(4): 604-609.

92.Chang, C. H., et al. (2010). "The usefulness of the laryngeal mask airway Fastrach for laryngeal surgery." EUROPEAN JOURNAL OF ANAESTHESIOLOGY 27(1): 20‐23.

93.Char, D. S., et al. (2012). "Case report: Airway and concurrent hemodynamic management in a neonate with oculo-auriculo-vertebral (Goldenhar) syndrome, severe cervical scoliosis, interrupted aortic arch, multiple ventricular septal defects, and an unstable cervical spine." Paediatric Anaesthesia 22(9): 932-934.

94.Chatterjee, D., et al. (2016). "Airway management in laryngotracheal injuries from blunt neck trauma in children." Paediatric Anaesthesia 26(2): 132-138.

95.Chaudhary, S., et al. (2023). ""ALL IN ONE": TRACHEAL STENOSIS, TRACHEOMALACIA, AND EXCESSIVE DYNAMIC AIRWAY COLLAPSE IN A PATIENT WITH AN ABERRANT INNOMINATE ARTERY PRESENTING IN ADULTHOOD." Chest 164(4): A5082-A5083.

96.Chaudhry, A., et al. (2019). "Cancer gone “nuts”." American Journal of Respiratory and Critical Care Medicine 199(9).

97.Chen, I. W., et al. (2022). "Comparison of video-stylet and conventional laryngoscope for endotracheal intubation in adults with cervical spine immobilization: A PRISMA-compliant meta-analysis." MEDICINE 101(33).

98.Chen, W. C., et al. (2022). "Case Report: Double Visualization Intubation Strategy for Patients With Ankylosing Spondylitis." FRONTIERS IN MEDICINE 9.

99.Cheng, T., et al. (2021). "Shikani Optical Stylet for Awake Nasal Intubation in Patients Undergoing Head and Neck Surgery." LARYNGOSCOPE 131(2): 319‐325.

100.Cheong, C. C., et al. (2023). "Partial vs full glottic view with CMAC(TM) D blade intubation of airway with simulated cervical spine injury: a randomized controlled trial." Expert Rev Med Devices 20(2): 151-160.

101.Chepanoske, M., et al. (2021). "Association between previous cervical spine fusion and failed first attempt at intubation: A retrospective chart review." JOURNAL OF NEUROSURGICAL ANESTHESIOLOGY 33(4): 383-384.

102ChiCtr (2020). "Novel Anesthesia technology of tracheal intubation in children with stenosed airway: a clinical trial."

103.ChiCtr (2023). "The clinical effect of fiberoptic bronchoscopy combined with video laryngoscope for nasotracheal intubation." https://trialsearch.who.int/Trial2.aspx?TrialID=ChiCTR2300073841.

104.ChiCtr (2023). "Effects of ultrasound-guided thoracic paravertebral nerve block combined with hydromorphone on postoperative analgesia and inflammatory response after thoracoscopy surgery." https://trialsearch.who.int/Trial2.aspx?TrialID=ChiCTR2300074082.

105.ChiCtr (2023). "Safe Easy Endotracheal Kit-flexible-guided endotracheal intubation reduces the stress response and postoperative airway complications under general anesthesia: a randomized controlled clinical study." https://trialsearch.who.int/Trial2.aspx?TrialID=ChiCTR2300067555.

106.Choi, S., et al. (2023). "Videolaryngoscope versus flexible fiberscope for orotracheal intubation in patients with cervical collar: a randomized controlled trial." Trends in Anaesthesia and Critical Care 48: S37.

107.Choi, S., et al. (2023). "Videolaryngoscopy vs. flexible fibrescopy for tracheal intubation in patients with cervical spine immobilisation: a randomised controlled trial." Anaesthesia 78(8): 970-978.

108.Choo, H. J., et al. (2015). "Educational suitability of endotracheal intubation using a video-laryngoscope." Korean journal of medical education 27(4): 267-274.

109.Chotai, P. N., et al. (2017). "Pediatric near-drowning events: do they warrant trauma team activation?" Journal of Surgical Research 212: 108-113.

110.Cohn, A. I. and M. H. Zornow (1995). "Awake endotracheal intubation in patients with cervical spine disease: A comparison of the Bullard laryngoscope and the fiberoptic bronchoscope." ANESTHESIA AND ANALGESIA 81(6): 1283-1286.

111.Cook, F., et al. (2019). "Prospective validation of a new airway management algorithm and predictive features of intubation difficulty." BRITISH JOURNAL OF ANAESTHESIA 122(2): 245-254.

112.Correa, J. B. B., et al. (2023). "Cervical spine movements during laryngoscopy and orotracheal intubation: A systematic review and meta-analysis." EMERGENCY MEDICINE JOURNAL 40(4): 300-307.

113.Cotton, B. A., et al. (2005). "Respiratory complications and mortality risk associated with thoracic spine injury." JOURNAL OF TRAUMA-INJURY INFECTION AND CRITICAL CARE 59(6): 1400-1407.

114.Crinquette, V., et al. (1994). "Techniques for intubation when head and neck cannot be moved." Agressologie: revue internationale de physio-biologie et de pharmacologie appliquées aux effets de l'agression 34 Spec No 1: 21-25.

115.Crosby, E. (2002). "Airway management after upper cervical spine injury: What have we learned?" Canadian Journal of Anesthesia 49(7): 733-744.

116.Crosby, E. T. (2006). "Airway management in adults after cervical spine trauma." Anesthesiology 104(6): 1293-1318.

117.Ctri (2014). "A COMPARISION OF LMA GUIDED FIBREOPTIC BRONCHOSCOPY AND VIDEOLARYNGOSCOPE FOR CERVICAL SPINE MOTION: WHICH IS BETTER??" https://trialsearch.who.int/Trial2.aspx?TrialID=CTRI/2014/10/005096.

118.Ctri (2017). "Measurement of upper neck movement with two different airway equipments used for airway protection." https://trialsearch.who.int/Trial2.aspx?TrialID=CTRI/2017/08/009433.

119.Cui, W., et al. (2023). "Difficult tracheostomy decannulation related to nasogastric tube syndrome: A case report." International Journal of Surgery Case Reports 110.

120.Cui, X. L., et al. (2013). "Comparative performance of GlideScope video laryngoscope and Macintosh laryngoscope in children with immobilized cervical spine." Pediatr Emerg Care 29(5): 690.

121.Czekajlo, M. S. and B. Zawada (2018). "Comparison of Intubrite and Macintosh laryngoscope use in immobilized cervical spine by novice nurses. A mannequin trial." Am J Emerg Med 36(5): 879-880.

122.Davidson, T. M., et al. (1975). "Endotracheal intubation with the flexible fiberoptic bronchoscope." EYE,EAR,NOSE THR.MONTHLY 54(9): 346-349.

123.Dawson, S. R., et al. (2020). "A controlled trial to investigate whether the orientation of the bevel and angle of approach determine the side of endobronchial intubation in an adult manikin." Journal of perioperative practice 30(3): 63‐68.

124.Delaney, J. S., et al. (2012). "The effect of laryngoscope handle size on possible endotracheal intubation success in University Football, Ice Hockey, and Soccer Players." CLINICAL JOURNAL OF SPORT MEDICINE 22(4): 341-348.

125.Desai, N., et al. (2019). "Comparative evaluation of Airtraq™ and GlideScope® videolaryngoscopes for difficult pediatric intubation in a Pierre Robin manikin." European journal of pediatrics 178(7): 1105‐1111.

126.Desamour, H. and A. Vincent (2022). "Cervical Dystonia and Anterior Cervical Spine Surgery." JOURNAL OF NEUROSURGICAL ANESTHESIOLOGY 34(4): 461-462.

127.Dey, A. K., et al. (2021). "A prospective, longitudinal study on effectiveness of king vision video assisted laryngoscope and mccoy laryngoscope in patients with simulated restricted neck mobility." Journal of Clinical and Diagnostic Research 15(3): UC21-UC24.

128.Dhar, S. I., et al. (2020). "Swallowing Characteristics in Zenker's-like Diverticulum After Anterior Cervical Spine Surgery." LARYNGOSCOPE 130(6): 1383-1387.

129.Dharanindra, M., et al. (2023). "Endotracheal Intubation with King Vision Video Laryngoscope vs Macintosh Direct Laryngoscope in ICU: A Comparative Evaluation of Performance and Outcomes." Indian Journal of Critical Care Medicine 27(2): 101-106.

130.Dhonneur, G., et al. (2016). "Face-to-face tracheal intubation with the video laryngoscope Airtraq in the sitting patients: A report of 3-year experience in patients after failed conventional tracheal intubation technique." Annals of intensive care 6.

131.Diwan, A. and S. Purohit (2019). "A Comparative Study of Orotracheal Intubation Guided by Airtraq and McCoy Laryngoscope in Patients with Rigid Cervical Collar In-situ Simulating Cervical Immobilization for Traumatic Cervical Spine Injury." Indian Journal of Neurosurgery 8(3): 161-167.

132.Dolder, P. and T. Gaszyński (2023). "ProVu™ video stylet in difficult airway management: case series." Trends in Anaesthesia and Critical Care 48: S30-S31.

133.Driver, B. E., et al. (2018). "Effect of use of a bougie vs endotracheal tube and stylet on first-attempt intubation success among patients with difficult airways undergoing emergency intubation a randomized clinical trial." JAMA - journal of the american medical association 319(21): 2179-2189.

134.Drks (2018). "Prospective randomized controlled trial for qualitative comparison between the double lumen tube and the bronchus blocker "EZ-blocker Endobronchial Blocker®" (Teleflex Life Sciences) for one-lung ventilation during thoracic surgery." https://trialsearch.who.int/Trial2.aspx?TrialID=DRKS00014816.

135.Dudda, M., et al. (2009). "Posttraumatic tracheal stenosis after complex fracture of the upper cervical spine : A rare complication." Unfallchirurg 112(8): 734-737.

136.Dullenkopf, A., et al. (2002). "Tracheal intubation in children with Morquio syndrome using the angulated video-intubation laryngoscope." Canadian Journal of Anesthesia 49(2): 198-202.

137.Dupanovic, M., et al. (2010). "Management of the airway in multitrauma." Current opinion in anaesthesiology 23(2): 276-282.

138.Durga, P., et al. (2012). "Comparison of tracheal intubation using the Airtraq® and Mc Coy laryngoscope in the presence of rigid cervical collar simulating cervical immobilisation for traumatic cervical spine injury." Indian Journal of Anaesthesia 56(6): 529-534.

139.Edens Th, E. and R. L. Sia (1981). "Flexible fiberoptic endoscopy in difficult intubations." Annals of Otology, Rhinology and Laryngology 90(4 I): 307-309.

140.Ein, S. H., et al. (1988). "Osteomyelitis of the cervical spine presenting as a neurenteric cyst." Journal of Pediatric Surgery 23(8): 779-781.

141.Eipe, N., et al. (2011). "The ankylosing spondylitis airway-between a rock and a hard place." Canadian Journal of Anesthesia 58: S125.

142.Ekka, M. and S. Sinha (2015). "Retropharyngeal abscess as a rare presentation of pulmonary tuberculosis." Lung India 32(3): 262-264.

143.Elazzazi, H. M., et al. (2020). "Bronchoscopy guided v.s ultrasound guided percutaneous tracheostomy." QJM 113(SUPPL 1): i18-i19.

144.Eldeyasty, B. K., et al. (2017). "Cervical spine motion during intubation: Comparing levitan fibreoptic stylet (FPS)® with macintosh laryngoscope." Canadian Journal of Anesthesia 64(1): S27.

145.El-Tahan, M. R., et al. (2017). "Cervical spine motion during tracheal intubation with King Vision™ video laryngoscopy and conventional laryngoscopy: a crossover randomized study." MINERVA ANESTESIOLOGICA 83(11): 1152-1160.

146.Elwood, T. and R. G. Cox (1996). "Laryngeal mask insertion with a laryngoscope in paediatric patients." Journal canadien d'anesthesie [Canadian journal of anaesthesia] 43(5 Pt 1): 435‐437.

147.Emmerich, K., et al. (2019). "Abstract 3: Comparison of Flight Physician operated Hyper-Angulated and Standard Geometry Video Laryngoscopy Tracheal Intubation on a Helicopter in a Manikin." Air medical journal 38(4): 259.

148.Estime, S. R. and C. M. Kuza (2019). "Trauma Airway Management: Induction Agents, Rapid Versus Slower Sequence Intubations, and Special Considerations." Anesthesiology Clinics 37(1): 33-50.

149.Falk, E. and A. Blumenberg (2023). "How to Build a Low-Cost Video-Assisted Laryngoscopy Suite for Airway Management Training." J Educ Teach Emerg Med 8(2): I1-i7.

150.Faraj, J. H. and T. Al Hamadi (2000). "Use of bullard laryngoscope in anesthesia for cervical spine surgery: Our experience in Qatar." Qatar Medical Journal 9(2): 33-35.

151.Farré, V., et al. (2014). "Modifications to the Airway Management Trainer Intubation Head for Training in Difficult Airway Recognition Through a Comparative Study With the Airsim<SUP>®</SUP> Intubation Head." SIMULATION IN HEALTHCARE-JOURNAL OF THE SOCIETY FOR SIMULATION IN HEALTHCARE 9(2): 136-140.

152.Farré, V., et al. (2014). "Modifications to the airway management trainer intubation head for training in difficult airway recognition through a comparative study with the Airsim® intubation head." Simulation in healthcare : journal of the Society for Simulation in Healthcare 9(2): 136-140.

153.Ferson, D. Z., et al. (2001). "Use of the Intubating LMA-Fastrach™ in 254 Patients with Difficult-to-manage Airways." Anesthesiology 95(5): 1175-1181.

154.Fiadjoe, J. E., et al. (2015). "A randomized multi-institutional crossover comparison of the GlideScope Cobalt Video laryngoscope to the flexible fiberoptic bronchoscope in a Pierre Robin manikin." Paediatric Anaesthesia 25(8): 801‐806.

155.Fiadjoe, J. E., et al. (2015). "A randomized multi-institutional crossover comparison of the GlideScope® Cobalt Video laryngoscope to the flexible fiberoptic bronchoscope in a Pierre Robin manikin." Paediatric Anaesthesia 25(8): 801‐806.

156.Fiadjoe, J. E., et al. (2009). "The efficacy of the storz miller 1 video laryngoscope in a simulated infant difficult intubation." ANESTHESIA AND ANALGESIA 108(6): 1783-1786.

157.Finfer, S. R., et al. (1989). "Cardiovascular responses to tracheal intubation: a comparison of direct laryngoscopy and fibreoptic intubation." ANAESTHESIA AND INTENSIVE CARE 17(1): 44‐48.

158.Foulds, L. T., et al. (2016). "A randomised cross-over trial comparing the McGrath<SUP>®</SUP> Series 5 videolaryngoscope with the Macintosh laryngoscope in patients with cervical spine immobilisation." Anaesthesia 71(4): 437-442.

159.Foulds, L. T., et al. (2016). "A randomised cross-over trial comparing the McGrath® Series 5 videolaryngoscope with the Macintosh laryngoscope in patients with cervical spine immobilisation." Anaesthesia 71(4): 437-442.

160.Fuchs, G., et al. (1999). "Fiberoptic intubation in 327 neurosurgical patients with lesions of the cervical spine." JOURNAL OF NEUROSURGICAL ANESTHESIOLOGY 11(1): 11-16.

161.Gadomski, B. C., et al. (2022). "Intubation biomechanics: Computational modeling to identify methods to minimize cervical spine motion and spinal cord strain during laryngoscopy and tracheal intubation in an intact cervical spine." JOURNAL OF CLINICAL ANESTHESIA 81.

162.Gadomski, B. C., et al. (2018). "Intubation biomechanics: validation of a finite element model of cervical spine motion during endotracheal intubation in intact and injured conditions." JOURNAL OF NEUROSURGERY-SPINE 28(1): 10-22.

163.Gadomski, B. C., et al. (2018). "Intubation biomechanics: Validation of a fnite element model of cervical spine motion during endotracheal intubation in intact and injured conditions." Journal of Neurosurgery: Spine 28(1): 10-22.

164.Gadomski, B. C., et al. (2017). "The effect of cervical spine injury on intervertebral kinetics and spinal cord strain during direct laryngoscopy: A computational investigation." Journal of Orthopaedic Research 35.

165.Gajraj, N. M., et al. (1994). "Cervical spine movement during orotracheal intubation: Comparison of the Belscope and Macintosh blades." Anaesthesia 49(9): 772-774.

166.Gaszynska, E., et al. (2013). "A comparison of conventional tube and EndoFlex tube for tracheal intubation in patients with a cervical spine immobilisation." Scandinavian journal of trauma, resuscitation and emergency medicine 21: 79.

167.Gawlowski, P. and L. Iskrzycki (2017). "Comparison of the KingVision video laryngoscope to the Macintosh laryngoscope for intubation of patient with cervical spine immobilization using extrication collars." Am J Emerg Med 35(4): 657-658.

168.Gawlowski, P., et al. (2017). "Comparison of the Macintosh laryngoscope and blind intubation via the iGEL for Intubation With C-spine immobilization: A Randomized, crossover, manikin trial." AMERICAN JOURNAL OF EMERGENCY MEDICINE 35(3): 484-487.

169.Ge, X., et al. (2021). "Evaluation of lightwand-guided endotracheal intubation for patients with missing or no teeth: a randomized controlled study." Brazilian journal of anesthesiology 71(4): 395‐401.

170.Gelaye, A., et al. (2014). "Difficult-to-wean: High index of suspicion." American Journal of Case Reports 15: 163-167.

171.Gercek, E., et al. (2008). "<i>In vivo</i> ultrasound real-time motion of the cervical spine during intubation under manual in-line stabilization:: a comparison of intubation methods." EUROPEAN JOURNAL OF ANAESTHESIOLOGY 25(1): 29-36.

172.Gercek, E., et al. (2008). "In vivo ultrasound real-time motion of the cervical spine during intubation under manual in-line stabilization: A comparison of intubation methods." EUROPEAN JOURNAL OF ANAESTHESIOLOGY 25(1): 29-36.

173.Gerling, M. C., et al. (2000). "Effects of cervical spine immobilization technique and laryngoscope blade selection on an unstable cervical spine in a cadaver model of intubation." ANNALS OF EMERGENCY MEDICINE 36(4): 293-300.

174.Gerstein, N. S., et al. (2010). "The fastrachTM intubating laryngeal mask airway®: An overview and update." Canadian Journal of Anesthesia 57(6): 588-601.

175.Ghaffar, W. B., et al. (2021). "Anaesthetic Challenges in a Paediatric Patient with Escobar Syndrome-Difficult Airway and Postoperative Pneumothorax." Turk J Anaesthesiol Reanim 49(6): 486-489.

176.Ghafoor, A. U., et al. (2005). "Caring for the patients with cervical spine injuries: What have we learned?" JOURNAL OF CLINICAL ANESTHESIA 17(8): 640-649.

177.Ghanem, M. T. and F. I. Ahmed (2017). "GlideScope versus McCoy laryngoscope: Intubation profile for cervically unstable patients in critical care setting." Egyptian journal of anaesthesia 33(1): 103-106.

178.Gill, N., et al. (2015). "Comparison of hemodynamic responses to intubation: Flexible fiberoptic bronchoscope versus McCoy laryngoscope in presence of rigid cervical collar simulating cervical immobilization for traumatic cervical spine." Anesth Essays Res 9(3): 337-342.

179.Gopalakrishnan, S., et al. (2008). "Anesthetic management of a patient with idiopathic arterial calcification of infancy and fused cervical spine." Paediatric Anaesthesia 18(10): 1006-1007.

180.Gottlieb, M., et al. (2016). "Utilization of a gum elastic bougie to facilitate single lung intubation." AMERICAN JOURNAL OF EMERGENCY MEDICINE 34(12): 2408‐2410.

181.Govil, D. and A. V. Pachisia (2022). "Debunk the Myth: Percutaneous Tracheostomy in Cervical Spine Injury." Indian Journal of Critical Care Medicine 26(10): 1067-1068.

182.Goyal, K., et al. (2017). "Comparison of polyvinyl chloride, curved reinforced, and straight reinforced endotracheal tubes for tracheal intubation through Airtraq™ laryngoscope in anesthetized patients." Journal of Anaesthesiology Clinical Pharmacology 33(3): 359-364.

183.Goyal, K. A., et al. (2017). "Comparison of polyvinyl chloride, curved reinforced, and straight reinforced endotracheal tubes for tracheal intubation through Airtraq™ laryngoscope in anesthetized patients." J Anaesthesiol Clin Pharmacol 33(3): 359-364.

184.Gravenstein, D., et al. (1999). "Clinical assessment of a plastic optical fiber stylet for human tracheal intubation." Anesthesiology 91(3): 648‐653.

185.Greenwood, M. (2004). "Laryngoscopy in emergency airway management." Trauma 46(3): 63-74.

186.Guinness, F., et al. (2022). "Dexmedetomidine sedation for a dental extraction in a patient with known difficult airway." Anaesthesia Reports 10(2).

187.Gulati, S., et al. (2022). "Comparison of Macintosh, McCoy, Truview EVO2, and King Vision Laryngoscopes for Intubation in Patients with Immobilized Cervical Spine: A Randomized, Controlled Trial." Bali Journal of Anesthesiology 6(2): 108-114.

188.Guo, X. and H. Ji (2022). "Management of Tracheal Perforation following Anterior Cervical Spine Surgery: Report of Two Cases and Review of the Literature." Case Rep Orthop 2022: 1914642.

189.Gupta, B., et al. (2014). "Topical airway anesthesia for awake fiberoptic intubation: Comparison between airway nerve blocks and nebulized lignocaine by ultrasonic nebulizer." Saudi J Anaesth 8(Suppl 1): S15-19.

190.Gupta, B., et al. (2014). "Topical airway anesthesia for awake fiberoptic intubation: Comparison between airway nerve blocks and nebulized lignocaine by ultrasonic nebulizer." Saudi journal of anaesthesia 8(5): S15-S19.

191.Gupta, N., et al. (2013). "Clinical evaluation of C-MAC videolaryngoscope with or without use of stylet for endotracheal intubation in patients with cervical spine immobilization." JOURNAL OF ANESTHESIA 27(5): 663-670.

192.Gupta, S. and R. S. Mane (2021). "Cmac videolaryngoscopy vs direct laryngoscopy: comparing the mouth-to-nose distance between the patient and laryngoscopist." Indian Journal of Critical Care Medicine 25(SUPPL 1): S87‐S88.

193.Gupta, S., et al. (2016). "Percutaneous tracheostomy in post-cervical spine fixation patients: Challenging the dogma." AMERICAN JOURNAL OF EMERGENCY MEDICINE 34(5): 910-911.

194.Hamano, N., et al. (2002). "Anesthesia for a severe ankylosing spondylitis patient whose posture had been restricted to only sitting for over 20 years." Japanese journal of anesthesiology 51(9): 1026-1028.

195.Hannoodi, F. and H. Sabbagh (2017). "ACE Inhibitor-Induced Angioedema following Cervical Spine Surgery." Case Reports in Cardiology 2017.

196.Haque, A., et al. (2012). "Percutaneous tracheostomy in patients with cervical spine immobilization." Neurocritical Care 17: S82.

197.Harhangi, B. S., et al. (2005). "Hemothorax as a complication after anterior cervical discectomy: case report." Neurosurgery 56(4): E871; discussion E871.

198.Harhangi, B. S., et al. (2005). "Hemothorax as a complication after anterior cervical discectomy: Case report." Neurosurgery 56(4): 871.

199.Hariharan, U., et al. (2014). "Airway management of fixed cervical spine undergoing transoral odontoidectomy: A novel technique." Current Medicine Research and Practice 4(4): 171-173.

200.Harrod, C. C., et al. (2010). "Epidural Pneumatosis of the Cervicothoracic Spine Associated With Transient Upper Motor Neuron Findings Complicating <i>Haemophilus influenzae</i> Pharyngitis, Bronchitis, and Mediastinitis." JOURNAL OF PEDIATRIC ORTHOPAEDICS 30(5): 455-459.

201.Hastings, R. H., et al. (1995). "Cervical spine movement during laryngoscopy with the Bullard, Macintosh, and Miller laryngoscopes." Anesthesiology 82(4): 859-869.

202.Hatano, K., et al. (1979). "Klippel-Feil syndrome and anesthesia. A case with spastic spinal paralysis." Japanese journal of anesthesiology 28(7): 733-737.

203.Hauswald, M., et al. (1991). "Cervical spine movement during airway management: Cinefluoroscopic appraisal in human cadavers." AMERICAN JOURNAL OF EMERGENCY MEDICINE 9(6): 535-538

204.Hazelton, J. P., et al. (2015). "The impact of a multidisciplinary safety checklist on adverse procedural events during bedside bronchoscopy-guided percutaneous tracheostomy." JOURNAL OF TRAUMA AND ACUTE CARE SURGERY 79(1): 111-116.

205.Hemmer, D., et al. (1982). "Intubation of a child with a cervical spine injury with the aid of a fiberoptic bronchoscope." ANAESTHESIA AND INTENSIVE CARE 10(2): 163-165.

206.Hikawa, Y., et al. (1994). "Use of Bullard intubating laryngoscope in emergency room." Japanese journal of anesthesiology 43(11): 1761-1765.

207.Hillel, A. T., et al. (2015). "A Novel Role for Otolaryngologists in the Multidisciplinary Difficult Airway Response Team." LARYNGOSCOPE 125(3): 640-644.

208.Hindman, B. J., et al. (2020). "Sex-Specific Intubation Biomechanics: Intubation Forces Are Greater in Male Than in Female Patients, Independent of Body Weight." Cureus 12(6): e8749.

209.Hindman, B. J., et al. (2022). "Relationship between Glottic View and Intubation Force during Macintosh and Airtraq Laryngoscopy and Intubation." ANESTHESIA AND ANALGESIA 135(4): 815-819.

210.Hindman, B. J., et al. (2016). "Intubation biomechanics: Laryngoscope force and cervical spine motion during intubation in cadavers - Effect of severe distractive-flexion injury on C3-4 motion." Journal of Neurosurgery: Spine 25(5): 545-555.

211.Hindman, B. J., et al. (2015). "Intubation Biomechanics." Anesthesiology 123(5): 1042-1058.

212.Hindman, B. J., et al. (2015). "Intubation Biomechanics: Laryngoscope Force and Cervical Spine Motion during Intubation in Cadavers-Cadavers versus Patients, the Effect of Repeated Intubations, and the Effect of Type II Odontoid Fracture on C1-C2 Motion." Anesthesiology 123(5): 1042-1058.

213.Hindman, B. J., et al. (2014). "Intubation Biomechanics <i>Laryngoscope Force and Cervical Spine Motion during Intubation with Macintosh and Airtraq Laryngoscopes</i>." Anesthesiology 121(2): 260-271.

214.Hindman, B. J., et al. (2014). "Intubation biomechanics: Laryngoscope force and cervical spine motion during intubation with macintosh and airtraq laryngoscopes." Anesthesiology 121(2): 260-271.

215.Hippard, H. K., et al. (2015). "A comparison between two videolaryngoscopes, the truview pcd and the glidescope cobolt AVL, in successfully intubating pediatric manikins with and without difficult airways." ANESTHESIA AND ANALGESIA 120(3): S279.

216.Hippard, H. K., et al. (2016). "A comparison of the Truview PCD and the GlideScope Cobalt AVL video-laryngoscopes to the Miller blade for successfully intubating manikins simulating normal and difficult pediatric airways." PEDIATRIC ANESTHESIA 26(6): 613-620.

217.Hippard, H. K., et al. (2016). "A comparison of the Truview PCD and the GlideScope Cobalt AVL video-laryngoscopes to the Miller blade for successfully intubating manikins simulating normal and difficult pediatric airways." Paediatric Anaesthesia 26(6): 613-620.

218.Hirabayashi, Y., et al. (2007). "Cervical spine movement during laryngoscopy using the airway scope compared with the macintosh laryngoscope." Anaesthesia 62(10): 1050-1055.

219.Hirabayashi, Y., et al. (2008). "A comparison of cervical spine movement during laryngoscopy using the Airtraq or Macintosh laryngoscopes." Anaesthesia 63(6): 635-640.

220.Hirabayashi, Y., et al. (2008). "A comparison of cervical spine movement during laryngoscopy using the Airtraq® or Macintosh laryngoscopes." Anaesthesia 63(6): 635-640.

221.Hirabayashi, Y., et al. (2010). "Distortion of anterior airway anatomy during laryngoscopy with the GlideScope videolaryngoscope." JOURNAL OF ANESTHESIA 24(3): 366-372.

222.Holmes, M. G., et al. (2018). "Airway management practice in adults with an unstable cervical spine: The harborview medical center experience." ANESTHESIA AND ANALGESIA 127(2): 450-454.

223.Hong, H., et al. (2019). "Study on the use of CT three-dimensional reconstruction technique for guiding tracheal intubation with rigid fiber bronchoscope in difficult airway." Beijing da xue xue bao. Yi xue ban = Journal of Peking University. Health sciences 51(5): 870-874.

224.Horton, W. A., et al. (1989). "Disposition of cervical vertebrae, atlanto-axial joint, hyoid and mandible during x-ray laryngoscopy." BRITISH JOURNAL OF ANAESTHESIA 63(4): 435-438.

225.Hosalli, V., et al. (2017). "Comparison of Airtraq™, McCoy™ and Macintosh laryngoscopes for endotracheal intubation in patients with cervical spine immobilisation: A randomised clinical trial." Indian Journal of Anaesthesia 61(4): 332-337.

226.Hossfeld, B., et al. (2016). "Out-of-hospital airway management in trauma patients. Experiences with the C-MAC® video laryngoscope." Unfallchirurg 119(6): 501-507.

227.Houde, B. J., et al. (2009). "A comparison of cervical spine motion during orotracheal intubation with the trachlight® or the flexible fiberoptic bronchoscope." ANESTHESIA AND ANALGESIA 108(5): 1638-1643.

228.Huang, W. T., et al. (2007). "Clinical comparisons between GlideScope video laryngoscope and Trachlight in simulated cervical spine instability." J Clin Anesth 19(2): 110-114.

229.Huang, W. T., et al. (2007). "Clinical comparisons between GlideScope® video laryngoscope and Trachlight® in simulated cervical spine instability." JOURNAL OF CLINICAL ANESTHESIA 19(2): 110-114.

230.Hughes, C. G., et al. (2010). "The McGrath video laryngoscope in unstable cervical spine surgery: a case series." J Clin Anesth 22(7): 575-576.

231.Hung, K. C., et al. (2021). "Comparison of video-stylet and video-laryngoscope for endotracheal intubation in adults with cervical neck immobilisation: A meta-analysis of randomised controlled trials." Anaesthesia Critical Care and Pain Medicine 40(6).

232.Hung, R. K., et al. (2013). "Does the use of a bougie reduce the force of laryngoscopy in a difficult airway with manual in-line stabilisation?: a randomised crossover simulation study." Eur J Anaesthesiol 30(9): 563-566.

233.Hung, R. K. Y., et al. (2013). "Does the use of a bougie reduce the force of laryngoscopy in a difficult airway with manual in-line stabilisation? :A randomised crossover simulation study." EUROPEAN JOURNAL OF ANAESTHESIOLOGY 30(9): 563-566.

234.Hung, R. K. Y., et al. (2013). "Does the use of a bougie reduce the force of laryngoscopy in a difficult airway with manual in-line stabilisation? <i>A randomised crossover simulation study</i>." EUROPEAN JOURNAL OF ANAESTHESIOLOGY 30(9): 563-566.

235.Hung, W. T., et al. (2001). "Evaluation of learning effectiveness in endotracheal intubation by the use of a laryngoscope in combination with a flexible fiberoptic bronchoscope." Acta anaesthesiologica Sinica 39(3): 129‐133.

236.Hwang, H., et al. (2010). "A case of distribution of pulmonary tuberculosis in bedridden patient with quadriplegia." American Journal of Respiratory and Critical Care Medicine 181(1).

237.Hyuga, S., et al. (2012). "Successful tracheal intubation with the McGrath(®) MAC video laryngoscope after failure with the Pentax-AWS™ in a patient with cervical spine immobilization." Can J Anaesth 59(12): 1154-1155.

238.Ihalainen, T., et al. (2018). "Risk factors for laryngeal penetration-aspiration in patients with acute traumatic cervical spinal cord injury." SPINE JOURNAL 18(1): 81-87.

239.Ilyas, S., et al. (2014). "A prospective randomised controlled trial comparing tracheal intubation plus manual in-line stabilisation of the cervical spine using the Macintosh laryngoscope vs the McGrath(®) Series 5 videolaryngoscope." Anaesthesia 69(12): 1345-1350.

240.Ilyas, S., et al. (2014). "A prospective randomised controlled trial comparing tracheal intubation plus manual in-line stabilisation of the cervical spine using the Macintosh laryngoscope vs the McGrath® Series 5 videolaryngoscope." Anaesthesia 69(12): 1345-1350.

241.İnan, G., et al. (2019). "Radiographic comparison of cervical spine motion using LMA fastrach, LMA Ctrach, and the macintosh laryngoscope." TURKISH JOURNAL OF MEDICAL SCIENCES 49(6): 1681-1686.

242.Iwai, C., et al. (2018). "Bilateral vocal cord palsy after a posterior cervical laminoplasty." EUROPEAN SPINE JOURNAL 27: S549-S554.

243.Jagannathan, N., et al. (2011). "Retrospective audit of the air-Q intubating laryngeal airway as a conduit for tracheal intubation in pediatric patients with a difficult airway." Paediatric Anaesthesia 21(4): 422-427.

244.Jain, D., et al. (2016). "Comparative effectiveness of McCoy laryngoscope and CMAC ® videolaryngoscope in simulated cervical spine injuries." Journal of Anaesthesiology Clinical Pharmacology 32(1): 59-64.

245.Jain, D., et al. (2014). "Comparison of the conventional CMAC and the D-blade CMAC with the direct laryngoscopes in simulated cervical spine injury-a manikin study." REVISTA BRASILEIRA DE ANESTESIOLOGIA 64(4): 269-274.

246.Jain, D., et al. (2014). "Comparison of the conventional CMAC and the D-blade CMAC with the direct laryngoscopes in simulated cervical spine injury--a manikin study." Braz J Anesthesiol 64(4): 269-274.

247.Jarrell, S. A., et al. (2015). "Paraplegia as initial presentation of sarcoidosis with neurological involvement." Journal of Investigative Medicine 63(2): 425.

248.Jayasinghe, A., et al. (2020). "Unstable cervical spine; is it still a contraindication for percutaneous tracheostomy as thought before?" Sri Lankan Journal of Anaesthesiology 28(2): 165-167.

249.Jenkins, K., et al. (2002). "Management choices for the difficult airway by anesthesiologists in Canada." Canadian Journal of Anesthesia 49(8): 850-856.

250.Jiang, A. A., et al. (2023). "Utilizing Airway Recording Function to Improve Pulmonary Critical Care Intubation Training." American Journal of Respiratory and Critical Care Medicine 207(1).

251.John, A. V., et al. (2017). "Development and testing of a low cost videolaryngoscope in a resource limited setting." Annals of Global Health 83(1): 4-5.

252.Jones, P. M., et al. (2009). "RCT: Glidescope cobalt vs regular glidescope for intubation." Canadian Journal of Anesthesia 56: S81.

253.Jun, D., et al. (2019). "Seizures and neurogenic pulmonary edema following moderate sedation and bronchoscopy." American Journal of Respiratory and Critical Care Medicine 199(9).

254.Jung, J. Y. (2015). "Airway management of patients with traumatic brain injury/C-spine injury." Korean Journal of Anesthesiology 68(3): 213-219.

255.Jurišić, I., et al. (2023). "AIRWAY MANAGEMENT WITH RIGID BRONCHOSCOPE IN ADULT PATIENT DURING CERVICAL SPINE SURGERY: A CASE REPORT." ACTA CLINICA CROATICA 62: 142-148.

256.Kamath, N., et al. (2020). "Comparison of ease of intubation with airtraq optical laryngoscope versus miller laryngoscope in neonates-a randomised interventional study." Indian Journal of Anaesthesia 64(13): S24-S25.

257.Kamga, H., et al. (2023). "Flexible nasal bronchoscopy vs. Airtraq® videolaryngoscopy for awake tracheal intubation: a randomised controlled non-inferiority study." Anaesthesia 78(8): 963‐969.

258.Kaplan, A., et al. (2016). "Comparison of the C-MAC Videolaryngoscope and Rigid Fiberscope with Direct Laryngoscopy in Easy and Difficult Airway Scenarios: A Manikin Study." JOURNAL OF EMERGENCY MEDICINE 50(3): e107-e114.

259.Kaplan, A., et al. (2016). "Comparison of the C-MAC Videolaryngoscope and Rigid Fiberscope with Direct Laryngoscopy in Easy and Difficult Airway Scenarios: A Manikin Study." J Emerg Med 50(3): e107-114.

260.Kapoor, R., et al. (2020). "Neuromonitoring as an alternative to awake intubation in uncooperative patients at risk for cervical spine injury." ACTA ANAESTHESIOLOGICA SCANDINAVICA 64(6): 863.

261.Karde, S., et al. (2018). "Cutaneous metastases as initial presentation of malignancy." BJR Case Rep 4(1): 20170059.

262.Kariya, T., et al. (2011). "Evaluation of the Pentax-AWS® and the Macintosh laryngoscope in difficult intubation: A manikin study." ACTA ANAESTHESIOLOGICA SCANDINAVICA 55(2): 223-227.

263.Katragadda, R., et al. (2022). "PRIMARY TRACHEAL SCHWANNOMA RESECTED WITH ELECTROCAUTERY SNARE." Chest 162(4): A1749.

264.Katsohiraki, M., et al. (2021). "Clinical evaluation of a patient with sublingual tracheal stenosis." Signa Vitae 17(5): 2.

265.Kawamoto, E., et al. (2010). "A case-control study of airway management for 68 patients with cervical spine injury: Comparison of the direct laryngoscope with a Macintosh blade and the fiberoptic bronchoscope." Japanese journal of anesthesiology 59(8): 976-980.

266.Kct (2018). "Comparison of two methods for placement of tube and confirmation of proper tube position during intubation using left-sided double-lumen endotracheal tube: fiberoptic bronchoscopy-guided method versus conventional intubation method using Macintosh laryngoscope." http://www.who.int/trialsearch/Trial2.aspx?TrialID=KCT0002663.

267.Keeffe, S. O. and D. O. Croinin (2023). "Diffuse Idiopathic Skeletal Hyperostosis: An Unusual cause of Recurrent Airway Obstruction." Journal of the Intensive Care Society 24(1): 65-66.

268.Keenan, M. A., et al. (1983). "Acquired laryngeal deviation associated with cervical spine disease in erosive polyarticular arthritis: Use of the fiberoptic bronchoscope in rheumatoid disease." Anesthesiology 58(5): 441-449.

269.Keller, C., et al. (1999). "Pressures exerted against the cervical vertebrae by the standard and intubating laryngeal mask airways: A randomized, controlled, cross-over study in fresh cadavers." ANESTHESIA AND ANALGESIA 89(5): 1296-1300.

270.Kendigelen, P., et al. (2016). "Emergency bronchoscopy for foreign-body aspiration in a child with type I mucopolysaccharidosis: a challenging airway management experience." JOURNAL OF ANESTHESIA 30(4): 696-698.

270.Khan, J. A. and A. Nileshwar (2009). "Comparison of the intubating laryngeal mask airway with the bullard laryngoscope for endotracheal intubation in patients with simulated difficult airway using the philadelphia cervical collar." Journal of Anaesthesiology Clinical Pharmacology 25(1): 33-37.

271.Khidr, A. M., et al. (2019). "Combined Use of a Fiberscope and Fuji Uniblocker for Removal of Retained Bronchial Tissue Glue After Repair of a Disrupted Left Main Bronchus." Seminars in Cardiothoracic and Vascular Anesthesia 23(3): 333-337.

272.Kienle, L. L., et al. (2023). "A novel 3D-printed laryngoscope with integrated working channels for laryngeal surgery." FRONTIERS IN SURGERY 10.

273.Kihara, S. and S. Watanabe (2001). "The intubating laryngeal mask: Its advantages and limitations." Japanese journal of anesthesiology 50(6): 604-612.

274.Kiliç, T., et al. (2013). "Upper cervical spine movement during intubation with different airway devices." AMERICAN JOURNAL OF EMERGENCY MEDICINE 31(7): 1034-1036.

275.Kılıç, T., et al. (2013). "Upper cervical spine movement during intubation with different airway devices." Am J Emerg Med 31(7): 1034-1036.

276.Kılıçaslan, A., et al. (2014). "Comparison of the C-MAC D-Blade, Conventional C-MAC, and Macintosh Laryngoscopes in Simulated Easy and Difficult Airways." Turk J Anaesthesiol Reanim 42(4): 182-189.

277.Kim, E., et al. (2016). "A comparison between the conventional and the laryngoscope-assisted lightwand intubation techniques in patients with cervical immobilization." Respirology 21: 142.

278.Kim, E., et al. (2017). "A Comparison between the Conventional and the Laryngoscope-Assisted Lightwand Intubation Techniques in Patients with Cervical Immobilization: A Prospective Randomized Study." ANESTHESIA AND ANALGESIA 125(3): 854-859.

279.Kim, H. J., et al. (2008). "Comparison of the GlideScope video laryngoscope and Macintosh laryngoscope in simulated tracheal intubation scenarios." EMERGENCY MEDICINE JOURNAL 25(5): 279-282.

280.Kim, H. Y., et al. (2019). "Comparison between use of single lightwand and video laryngoscope-guided lightwand for tracheal intubation in simulated cervical spine-immobilized patients: a single-blind randomized study." JOURNAL OF INTERNATIONAL MEDICAL RESEARCH 47(11): 5632-5642.

281.Kim, H. Y., et al. (2019). "Comparison between use of single lightwand and video laryngoscope-guided lightwand for tracheal intubation in simulated cervical spine-immobilized patients: a single-blind randomized study." JOURNAL OF INTERNATIONAL MEDICAL RESEARCH 47(11): 5632-5642.

282.Kim, J. H., et al. (2014). "Comparison of three types of intubation stylets for tracheal intubation with McGRATH MAC video laryngoscope by novice intubators in a simulated cervical spine immobilization: A randomized crossover manikin study." RESUSCITATION 85: S27-S28.

283.Kim, J. Y., et al. (2022). "Fiberoptic-guided nerve integrity monitoring tube intubation assisted by video-laryngoscope with external laryngeal manipulation in a patient with anteriorly displaced larynx due to huge goiter with retropharyngeal involvement: A case report." Medicine (United States) 101(10): E29041.

284.Kim, T. K., et al. (2017). "A Randomized Crossover Study Comparing Cervical Spine Motion during Intubation between Two Lightwand Intubation Techniques in Patients with Simulated Cervical Immobilization: Laryngoscope-Assisted Versus Conventional Lightwand Intubation." ANESTHESIA AND ANALGESIA 125(2): 485-490.

285.Kini, G., et al. (2008). "Comparison of two techniques for insertion of classic laryngeal mask airway." Journal of anaesthesiology, clinical pharmacology 24(4): 441‐443.

286.Kita, S., et al. (2014). "Head extension during laryngoscopy for obtaining a best glottic view: Comparison of the McGrath® and MacIntosh laryngoscopes." Japanese journal of anesthesiology 63(12): 1300-1305.

287.Kita, S., et al. (2014). "[Head extension during laryngoscopy for obtaining a best glottic view: comparison of the McGrath and Macintosh laryngoscopes]." Masui 63(12): 1300-1305.

288.Ko, J. I., et al. (2015). "Comparison of intubation times using a manikin with an immobilized cervical spine: Macintosh laryngoscope vs. GlideScope vs. fiberoptic bronchoscope." Clin Exp Emerg Med 2(4): 244-249.

289.Kok, T., et al. (2012). "Effectiveness and safety of the Levitan FPS Scope™ for tracheal intubation under general anesthesia with a simulated difficult airway." Canadian Journal of Anesthesia 59(8): 743-750.

290.Kokita, A., et al. (2023). "A case of safe airway management by fiber-optic nasotracheal intubation in general anesthesia in a pediatric patient with Hajdu-Cheney syndrome: a case report." JA Clinical Reports 9(1).

291.Komatsu, R., et al. (2009). "Airway Scope and StyletScope for Tracheal Intubation in a Simulated Difficult Airway." ANESTHESIA AND ANALGESIA 108(1): 273-279.

292.Konishi, A., et al. (1998). "Cervival spine movement during light-guided orotracheal intubation with lightwand stylet (Trachlight)." Japanese journal of anesthesiology 47(1): 94-97.

293.Konishi, A., et al. (1997). "Cervical spine movement during orotracheal intubation using the McCoy laryngoscope compared with the Macintosh and the Miller laryngoscopes." Japanese journal of anesthesiology 46(1): 124-127.

294.Korkusuz, M., et al. (2023). "The effect of the use of a stylet and/or videolaryngoscope on intubation time in obese patients: a randomized clinical trial." Trends in Anaesthesia and Critical Care 48: S13-S15.

295.Kovitwanawong, N. and L. Suwansukho (2013). "A comparison of cervical spine movement during tracheal intubation when using a Pentax Airway Scope or the GlideScope video laryngoscope with fluoroscopy." JOURNAL OF CLINICAL ANESTHESIA 25(3): 251.

296.Koyama, Y. and T. Andoh (2018). "Evaluation of effective emergency airway management for accidental extubation in a patient with head fixed in the prone position: A randomized crossover manikin study." ANESTHESIA AND ANALGESIA 126(4): 12.

297.Krafft, P. and M. Frass (2000). "The difficult airway." WIENER KLINISCHE WOCHENSCHRIFT 112(6): 260-270.

298.Kreitz, A., et al. (2013). "Awake videolaryngoscopic intubation in a patient with a penetrating neck injury." Canadian Journal of Anesthesia 60(1): S38.

299.Kriege, M., et al. (2015). "Comparison of GlideScope®Cobalt and McGrath® Series 5 video laryngoscopes with direct laryngoscopy in a simulated regurgitation/aspiration scenario." Medizinische klinik - intensivmedizin und notfallmedizin 110(3): 218-224.

300.Kriege, M., et al. (2015). "Comparison of GlideScopeA® Cobalt and McGrathA®A Series 5 video laryngoscopes with direct laryngoscopy in a simulated regurgitation/aspiration scenario." MEDIZINISCHE KLINIK-INTENSIVMEDIZIN UND NOTFALLMEDIZIN 110(3): 218-224.

301.Krochmal, R., et al. (2014). "Tracheobronchial stenosis as a result of chronic silent aspiration." American Journal of Respiratory and Critical Care Medicine 189.

302.Kumar, D., et al. (2019). "GlideScope versus D-blade for tracheal intubation in cervical spine patients: A randomised controlled trial." Indian J Anaesth 63(7): 544-550.

303.Kumar, N., et al. (2015). "Airway management in a patient of ankylosing spondylitis with traumatic cervical spine injury." Saudi journal of anaesthesia 9(3): 327-329.

304.Kumar, S. and P. Singh (2020). "SWIFT ACUTE EMERGENCY PERSONAL NOVEL VIDEO LARYNGOSCOPE (SAPNOSCOPE): AN INNOVATIVE SOLUTION AND POTENTIAL GAME-CHANGER ON THE HORIZON FOR INTUBATION IN RESOURCE-LIMITED COUNTRIES." Chest 158(4): A2443.

305.Kumari, A., et al. (2023). "A comparative study of the C-MAC D-blade videolaryngoscope and McCoy laryngoscope for oro-tracheal intubation with manual in-line stabilization of neck in patients undergoing cervical spine surgery." Journal of Anaesthesiology Clinical Pharmacology 39(3): 435-443.

306.Kunisch-Hoppe, M., et al. (2000). "Tracheal rupture caused by blunt chest trauma: Radiological and clinical features." EUROPEAN RADIOLOGY 10(3): 480-483.

307.Lakemeier, S., et al. (2009). "Odontoid process metastasis of bronchial carcinoma as a rare cause for nonmechanical neck pain: A case report." Cases journal 2(6).

308.Langenstein, H. and G. Cunitz (1996). "Difficult intubation in adults." Anaesthesist 45(4): 372-383.

309.Langeron, O., et al. (1992). "Endotracheal intubation of patients with cervical spine injury using a fiberoptic laryngoscope." Annales francaises d'anesthesie et de reanimation 11(3): 388-391.

310.Langeron, O., et al. (1992). "[Tracheal intubation in patients with cervical spine injuries using a fiber optic laryngoscope]." Ann Fr Anesth Reanim 11(3): 388-391.

311.Laosuwan, P., et al. (2015). "Randomized cinefluoroscopic comparison of cervical spine motion using McGrath series 5 and Macintosh laryngoscope for intubation with manual in-line stabilization." Journal of the Medical Association of Thailand 98: S63-S69.

312.Latorre, F., et al. (1993). "Stress response to nasotracheal intubation. A comparative evaluation of fiberendoscopic vs. laryngoscopic intubation with and without topical anaesthesia of the larynx. FIBEROPTISCHE INTUBATION UND STRESS." Der Anaesthesist 42(7): 423‐426.

313.Laurent, S. C., et al. (1996). "The use of the McCoy laryngoscope in patients with simulated cervical spine injuries." Anaesthesia 51(1): 74-75.

314.Laus, M., et al. (1993). "Anterior decompression and plate fixation in fracture dislocations of the lower cervical spine." EUROPEAN SPINE JOURNAL 2(2): 82-88.

315.Lee, I. Y. C., et al. (2010). "Comparison of the Karl Storz video laryngoscope with the Macintosh laryngoscope for intubating difficult airway: a manikin study." HONG KONG JOURNAL OF EMERGENCY MEDICINE 17(4): 332-340.

316.Lee, J. J., et al. (2012). "Fiberoptic intubation through a laryngeal mask airway as a management of difficult airwary due to the fusion of the entire cervical spine - A report of two cases." Korean Journal of Anesthesiology 62(3): 272-276.

317.Lee, K. W., et al. (2012). "2011 National Football League RSI survey." ANNALS OF EMERGENCY MEDICINE 60(4): S66.

318.Lee, Y. C. I., et al. (2010). "Comparison of the Karl Storz video laryngoscope with the Macintosh laryngoscope for intubating difficult airway: A manikin study." HONG KONG JOURNAL OF EMERGENCY MEDICINE 17(4): 332-340.

319.Leiva-Juarez, M. M., et al. (2018). "Migration of spinal pedicle screw with aerodigestive perforation and subsequent device expectoration." International Journal of Surgery Case Reports 46: 6-8.

340.

341.Lenhardt, R., et al. (2014). "Is video laryngoscope-assisted flexible tracheoscope intubation feasible for patients with predicted difficult airway? A prospective, randomized clinical trial." ANESTHESIA AND ANALGESIA 118(6): 1259-1265.

342.Levy-Faber, D., et al. (2015). "Comparison of VivaSight double-lumen tube with a conventional double-lumen tube in adult patients undergoing video-assisted thoracoscopic surgery." Anaesthesia. 70 (11) (pp 1259-1263), 2015. Date of publication: november 2015.

343.Li, X. Y., et al. (2007). "Comparison of hemodynamic responses to nasotracheal intubations with Glide Scope video-laryngoscope, Macintosh direct laryngoscope, and fiberoptic bronchoscope." Zhongguo yi xue ke xue yuan xue bao. Acta Academiae Medicinae Sinicae 29(1): 117‐123.

344.Liao, S., et al. (2017). "Cadaveric study of movement in the unstable upper cervical spine during emergency management: Tracheal intubation and cervical spine immobilisation - A study protocol for a prospective randomised crossover trial." BMJ Open 7(8).

345.Liberale, C., et al. (2023). "Step-By-Step Surgery for Diffuse Idiopathic Skeletal Hyperostosis (DISH) of the Cervical Spine." LARYNGOSCOPE.

346.Lim, J. A., et al. (2020). "The Effect of Fiberoptic Bronchoscopy-guided Technique for Placement of a Left-sided Double-lumen Tube on the Intubation Performance Compared with the Conventional Method Using a Macintosh Laryngoscope." Open anesthesia journal 14: 115‐122.

347.Liu, C. H., et al. (2015). "Airway obstruction due to tracheomalacia caused by innominate artery compression and a kyphotic cervical spine." ANNALS OF THORACIC SURGERY 99(2): 685-687.

348.Liu, E. H., et al. (2008). "The Airway Scope, a new video laryngoscope: its use in three patients with cervical spine problems." Br J Anaesth 100(1): 142-143.

349.Liu, L., et al. (2010). "Tracheal intubation of a difficult airway using airway scope, airtraq, and macintosh laryngoscope: A comparative manikin study of inexperienced personnel." ANESTHESIA AND ANALGESIA 110(4): 1049-1055.

350.Liu, L. J., et al. (2010). "Tracheal Intubation of a Difficult Airway Using Airway Scope, Airtraq, and Macintosh Laryngoscope: A Comparative Manikin Study of Inexperienced Personnel." ANESTHESIA AND ANALGESIA 110(4): 1049-1055.

351.Liu, W. F., et al. (2012). "Effects of different nasotracheal intubations in obstructive sleep apnea hypopnea syndrome patients with uvulopalatopharyngoplasty." Zhonghua Yi Xue Za Zhi 92(43): 3067‐3071.

352.Liu, X., et al. (2022). "Comparative analysis of popular predictors for difficult laryngoscopy using hybrid intelligent detection methods." Heliyon 8(11): e11761.

353.Long, C., et al. (2016). "Planning airway management in a previous failed intubation." Anaesthesia 71: 11.

354.Lopez Mock, C., et al. (2017). "Kingvision videolaryngoscope versus macintosh laryngoscope: Comparative study in patients with manual in line stabilization." JOURNAL OF NEUROSURGICAL ANESTHESIOLOGY 29(4): 530-532.

355.Lu, A., et al. (2022). "eP179: Expanding the phenotype of CLCN6-associated early-onset neurodegeneration." Genetics in Medicine 24(3): S108-S111.

356.Lütcke, S., et al. (2011). "Pictorial representations of orotracheal intubation in the medical literature." Anasthesiologie und Intensivmedizin 52(9): 681-692

357.MacIntyre, P. A., et al. (1999). "Cervical spine movements during laryngoscopy. Comparison of the Macintosh and McCoy laryngoscope blades." Anaesthesia 54(5): 413-418.

358.MacIntyre, P. A., et al. (1999). "Cervical spine movements during laryngoscopy - Comparison of the Macintosh and McCoy laryngoscope blades." Anaesthesia 54(5): 413-418.

359.MacIntyre, P. A., et al. (1999). "Cervical spine movements during laryngoscopy. Comparison of the Macintosh and McCoy laryngoscope blades." Anaesthesia 54(5): 413-418.

360.Macko, C. A. (2022). "Chest X-ray Unremarkable: A Case of a 'Late Blooming' Empyema." American Journal of Respiratory and Critical Care Medicine 205(1).

361.MacQuarrie, K., et al. (1999). "Tracheal intubation using a Bullard laryngoscope for patients with a simulated difficult airway." Canadian Journal of Anaesthesia 46(8): 760-765.

362.MacQuarrie, K., et al. (1999). "Tracheal intubation using a Bullard laryngoscope for patients with a simulated difficult airway." CANADIAN JOURNAL OF ANAESTHESIA-JOURNAL CANADIEN D ANESTHESIE 46(8): 760-765.

363.MacQuarrie, K., et al. (1999). "Tracheal intubation using Bullard laryngoscope for patients with a simulated difficult airway." Can J Anaesth 46(8): 760-765.

364.Madhavan, A., et al. (2023). "Respiratory Failure Now After Remote History Of Neck Surgery." American Journal of Respiratory and Critical Care Medicine 207(1).

365.Madziala, M., et al. (2017). "Comparison of direct versus video-laryngoscopy during simulated pediatric cervical spine immobilization." Pediatria polska 92(4): 406-411.

366.Mahadevaiah, T., et al. (2022). "Comparison of Hemodynamic Response to Laryngoscopy Using Miller and McCoy Blade." Cureus 14(5): e24914.

367.Maharaj, C. H., et al. (2007). "Endotracheal intubation in patients with cervical spine immobilization - A comparison of Macintosh and Airtraq laryngoscopes." Anesthesiology 107(1): 53-59.

368.Maharaj, C. H., et al. (2007). "Endotracheal intubation in patients with cervical spine immobilization: A comparison of Macintosh and Airtraq laryngoscopes." Anesthesiology 107(1): 53-59.

369.Mahmoudpour, A., et al. (2007). "Awake tracheal intubation via intubating laryngeal mask vs direct laryngoscopy and cervical spine excursion." PAKISTAN JOURNAL OF MEDICAL SCIENCES 23(2): 238-241.

370.Mahmoudpour, A., et al. (2007). "A wake tracheal intubation via intubating laryngeal mask vs direct laryngoscopy and cervical spine excursion." PAKISTAN JOURNAL OF MEDICAL SCIENCES 23(2): 238-241.

371.Mahrous, R. S. S. and A. M. M. Ahmed (2018). "The Shikani Optical Stylet as an Alternative to Awake Fiberoptic Intubation in Patients at Risk of Secondary Cervical Spine Injury: A Randomized Controlled Trial." JOURNAL OF NEUROSURGICAL ANESTHESIOLOGY 30(4): 354-358.

372.Majernick, T. G., et al. (1986). "Cervical spine movement during orotracheal intubation." ANNALS OF EMERGENCY MEDICINE 15(4): 417-420.

373.Malcharek, M. J., et al. (2014). "Comparison of Aintree and Fastrach techniques for low- skill fibreoptic intubation in patients at risk of secondary cervical injury." EUROPEAN JOURNAL OF ANAESTHESIOLOGY 31(3): 153-158.

374.Malcharek, M. J., et al. (2014). "Comparison of Aintree and Fastrach techniques for low-skill fibreoptic intubation in patients at risk of secondary cervical injury: A randomised controlled trial." EUROPEAN JOURNAL OF ANAESTHESIOLOGY 31(3): 153-158.

375.Maldini, B., et al. (2016). "Challenges in the use of video laryngoscopes." Acta Clinica Croatica, Supplement 55: 41-50.

376.Maldonado, I., et al. (2009). "Safety of percutaneous tracheostomy in trauma patients." Critical care medicine 37(12): A70.

377.Maldonado, I. M. and S. B. Becker (2010). "Safety of percutaneous tracheostomy in trauma patients." CRITICAL CARE 14: S78.

378.Malik, M. A., et al. (2008). "Comparison of Macintosh, Truview EVO2, Glidescope, and Airwayscope laryngoscope use in patients with cervical spine immobilization." Br J Anaesth 101(5): 723-730.

379.Malik, M. A., et al. (2008). "Comparison of Macintosh, Truview EVO2®, Glidescope®, and Airwayscope® laryngoscope use in patients with cervical spine immobilization." BRITISH JOURNAL OF ANAESTHESIA 101(5): 723-730.

380.Malik, M. A., et al. (2009). "Tracheal intubation in patients with cervical spine immobilization: a comparison of the Airwayscope, LMA CTrach, and the Macintosh laryngoscopes." Br J Anaesth 102(5): 654-661.

381.Malik, M. A., et al. (2009). "Tracheal intubation in patients with cervical spine immobilization: A comparison of the Airwayscope®, LMA CTrach®, and the macintosh laryngoscopes." BRITISH JOURNAL OF ANAESTHESIA 102(5): 654-661.

382.Malik, S., et al. (2014). "Use of airtraq optical laryngoscope for naso-tracheal intubation in anaesthetized patients." Acta Medica International 1(1): 41-42.

383.Manikandan, S., et al. (2010). "Ultrasound-guided bilateral superior laryngeal nerve block to aid awake endotracheal intubation in a patient with cervical spine disease for emergency surgery." ANAESTHESIA AND INTENSIVE CARE 38(5): 946-948.

384.Manlapaz, M., et al. (2022). "Airway Management in Cervical Spine Pathologies." Current Anesthesiology Reports 12(3): 373-381.

385.Manninen, P. H., et al. (2007). "Management of the airway in patients undergoing cervical spine surgery." JOURNAL OF NEUROSURGICAL ANESTHESIOLOGY 19(3): 190-194.

386.Manoach, S. and L. Palladino (2007). "Manual in-line stabilization for acute airway management of suspected cervical spine injury: Historical review and current questions." ANNALS OF EMERGENCY MEDICINE 50(3): 236-245.

387.Maremanda, K. R., et al. (2023). "Comparison of Intubation Conditions Between Airtraq, McGrath Video Laryngoscopes, and Macintosh Under Conditions of Simulated Trauma Airway and Rapid Sequence Induction Intubation." JOURNAL OF EMERGENCY MEDICINE 64(3): 271-281.

388.Márquez, X. G. and A. E. Márquez (2002). "The bullard laryngoscope as an alternative in difficult airway management. A review." Difficult Airways 3(1): 8-14.

389.Martinez, A. E., et al. (1993). "Comparison between cricothyroid injection and laryngoscopic as methods for delivering lidocaine in bronchoscopy." European respiratory journal. Supplement. 6 Suppl 17: 546S.

390.Martínez Parra, L. M., et al. (2016). "Dexmedetomidine an alternative medication for awake fiberoptic intubation in a patient with C2 axis fracture." JOURNAL OF NEUROSURGICAL ANESTHESIOLOGY 28(2): S3.

391.Martinez-Hurtado, E., et al. (2020). "Airway management with IntuBrite laryngoscope device disposable blades. About a case serie." Trends in Anaesthesia and Critical Care 30: e88.

392.Martínez-Ruiz, Y. I. and J. Vázquez-Torres (2017). "Airway in the polytraumatized patient: Use of videolaryngoscopy as an alternative and solution." Revista Mexicana de Anestesiologia 40(2): 113-119.

393.Maruyama, K., et al. (2008). "Randomized cross-over comparison of cervical-spine motion with the AirWay Scope or Macintosh laryngoscope with in-line stabilization: A video-fluoroscopic study." BRITISH JOURNAL OF ANAESTHESIA 101(4): 563-567.

394.Maruyama, K., et al. (2008). "Upper cervical spine movement during intubation: Fluoroscopic comparison of the AirWay Scope, McCoy laryngoscope, and Macintosh laryngoscope." BRITISH JOURNAL OF ANAESTHESIA 100(1): 120-124.

395.Mastanduno, S., et al. (2019). "Cervical brown-sequard syndrome due to neurosarcoidosis in a patient with HIV: A case report." PM and R 11: S159-S160.

396.Masui, D., et al. (2022). "Negative-Pressure Pulmonary Edema After Difficult Endotracheal Intubation in a Patient with Juvenile Rheumatoid Arthritis Undergoing Spigelian Hernia Surgery: A Case Report." American Journal of Case Reports 23.

397.Mathew, D. G., et al. (2014). "Endotracheal intubation with Intubating Laryngeal Mask Airway (ILMA)™, C-Trach™, and Cobra PLA™ in simulated cervical spine injury patients: a comparative study." JOURNAL OF ANESTHESIA 28(5): 655-661.

398.Mathew, N., et al. (2018). "Comparison of haemodynamic responses to tracheal intubation using macintosh and airtraq® laryngoscope in patients with simulated cervical spine injury." Sri Lankan Journal of Anaesthesiology 26(2): 124-130.

399.Mathew, S., et al. (2013). "Airway management in Escobar syndrome: A formidable challenge." Indian Journal of Anaesthesia 57(6): 603-605.

400.Matos, L., et al. (2021). "Management of an anticipated difficult airway in Hunter's syndrome." ANESTHESIA AND ANALGESIA 133(3 SUPPL 2): 1259.

401.McCann, A. C., et al. (2019). "Novel approach using transoral robotic surgery for resection of cervical spine chordoma." LARYNGOSCOPE 129(6): 1395-1399.

402.McClelland, S., et al. (2020). "Guillain-barre syndrome or is it?" Journal of Investigative Medicine 68(2): 604.

403.McElwain, J. and J. G. Laffey (2011). "Comparison of the C-MAC®, Airtraq®, and Macintosh laryngoscopes in patients undergoing tracheal intubation with cervical spine immobilization." BRITISH JOURNAL OF ANAESTHESIA 107(2): 258-264.

404.Meena, R. K., et al. (2022). "Trans-Oesophageal Migration of Pulled-Out Locking Screw (Zero-Profile Implant System) and its Retrieval Using Suction Catheter: A Technical Note." NEUROLOGY INDIA 70(2): 749-752.

405.Meleca, J. B., et al. (2021). "Anterolateral Thigh Fascia Lata Rescue Flap: A New Weapon in the Battle Against Osteoradionecrosis." LARYNGOSCOPE 131(12): 2688-2693.

406.Mendonça, M., et al. (2021). "When past is part of the future-an airway management approach in cervical spine surgery from a Neurosurgical Center." ANESTHESIA AND ANALGESIA 133(3 SUPPL 2): 939.

407.Mentzelopoulos, S. D., et al. (2000). "Balloon laryngoscopy reduces head extension and blade leverage in patients with potential cervical spine injury." CRITICAL CARE 4(1): 40-44.

408.Mercer, M. H. and D. A. Gabbott (1998). "Insertion of the combination airway with the cervical spine immobilised in a rigid cervical collar." Anaesthesia 53(10): 971-974.

409.Mercer, M. H. and D. A. Gabbott (1998). "Insertion of the Combitube airway with the cervical spine immobilised in a rigid cervical collar." Anaesthesia 53(10): 971-974.

410.Meschino, A., et al. (1992). "The safety of awake tracheal intubation in cervical spine injury." Canadian Journal of Anaesthesia 39(2): 114-117.

411.Moda, N. and N. Kumar (2018). "Combined use of video laryngoscopy and fiberoptic for airway management in a patient with fixed cervical spine." Asian Journal of Pharmaceutical and Clinical Research 11(11): 1-3.

412.Mohamad Zaini, R. H., et al. (2016). "Comparison of the effectiveness between C-Mac D-Blade and glidescope ranger for tracheal intubation in simulated patient with cervical spine immobilisation." ANESTHESIA AND ANALGESIA 123(3): 695.

413.Mohamed, N. N., et al. (2014). "Effect of fiberoptic intubation on myocardial ischemia and hormonal stress response in diabetics with ischemic heart disease." Egyptian journal of anaesthesia 30(1): 53‐58.

414.Moschini, V., et al. (1999). "The use of bronchial fibroscopy for difficult intubations in maxillofacial surgery." MINERVA ANESTESIOLOGICA 65(12): 843-847.

415.Mounika, K., et al. (2022). "Comparison of Airtraq DL ™ and Macintosh laryngoscope for double-lumen tube placement in simulated difficult airway: a randomised study." Indian Journal of Anaesthesia 66(6): 442‐448.

416.Mulder, D. S., et al. (1975). "The use of the fiberoptic bronchoscope to facilitate endotracheal intubation following head and neck trauma." Journal of trauma 15(8): 638-640.

417.Muldoon, S., et al. (2020). "The challenge of airway management in patients with “chin-on-chest” deformities undergoing spinal fixation." Trends in Anaesthesia and Critical Care 30: e130.

418.Mullen, T., et al. (2012). "Comparing efficacy of king vision and glidescope in cadavers." Canadian Journal of Anesthesia 59.

419.Murphy, L. D., et al. (2014). "Comparison of the king vision video laryngoscope with the Macintosh laryngoscope." JOURNAL OF EMERGENCY MEDICINE 47(2): 239-246.

420.Nair, S., et al. (2021). "Comparison of performance characteristics of C-MAC video, McCoy, and Macintosh laryngoscopes in elective cervical spine surgery." Journal of Anaesthesiology Clinical Pharmacology 37(4): 569-573.

421.Nair, S. M., et al. (2021). "Comparison of performance characteristics of C-MAC video, McCoy, and Macintosh laryngoscopes in elective cervical spine surgery." J Anaesthesiol Clin Pharmacol 37(4): 569-573.

422.Nakayama, Y., et al. (2010). "An evaluation of double lumen tube placement using airway scope, glidescope or macintosh laryngoscope." ANESTHESIA AND ANALGESIA 110(3): S258.

423.Nakazawa, K., et al. (1999). "Using the intubating laryngeal mask airway (LMA-Fastrach(TM)) for blind endotracheal intubation in patients undergoing cervical spine operation." ANESTHESIA AND ANALGESIA 89(5): 1319-1321.

424.Nandadeva, D., et al. (2012). "Unilateral diaphragmatic paralysis: How far to investigate?" Respirology 17: 60.

425."Comparison of the Glidescope and Pentax AWS laryngoscopes to the Macintosh laryngoscope for use by advanced paramedics in easy and simulated difficult intubation." BMC Emerg Med 9: 9.

426.Nasim, S., et al. (2009). "Comparison of the Glidescope® and Pentax AWS® laryngoscopes to the Macintosh laryngoscope for use by Advanced Paramedics in easy and simulated difficult intubation." BMC emergency medicine 9.

427.Nct (2018). "The Comparison of Awake Fiberoptic And Awake Video Laryngoscopy Tracheal Intubation in Cervical Surgery." https://clinicaltrials.gov/show/NCT03734965.

428.Nct (2021). "Macintosh Versus GlideScope Versus C-MAC for Double Lumen Endotracheal Intubation." https://clinicaltrials.gov/show/NCT05091281.

429.Nct (2021). "Neutral Position Facilitates Orotracheal Intubation With Videolaryngoscopes." https://clinicaltrials.gov/show/NCT04858906.

430.Nct (2022). "The Effects of Individualized Lung-protective Ventilation With Lung Dynamic Compliance-guided Positive End-expiratory Pressure(PEEP) Titration on Postoperative Pulmonary Complications of Pediatric Video-assisted Thoracoscopic Surgery." https://clinicaltrials.gov/show/NCT05386901.

431.Nct (2023). "ATP and P2X3 Receptor in Chronic Cough." https://clinicaltrials.gov/show/NCT05713019.

432.Nct (2023). "Combined Use of Glidescope With Fiber Optic Broncscopy Versus Fiber Optic Alone in Difficult Intubation." https://clinicaltrials.gov/show/NCT05751590.

433.Nct (2023). "Hybrid Intubation Technique for Difficult Airway Children." https://clinicaltrials.gov/ct2/show/NCT06058221.

434.Ni, X., et al. (2013). "Treatment of cervical vertebral (C1) metastasis of lung cancer with radiotherapy: A case report." Oncology letters 5(4): 1129-1132.

435.Nileshwar, A. and V. Garg (2010). "Comparison of Bullard laryngoscope and short-handled Macintosh laryngoscope for orotracheal intubation in pediatric patients with simulated restriction of cervical spine movements." Paediatric Anaesthesia 20(12): 1092-1097.

436.Nileshwar, A. and A. Thudamaladinne (2007). "Comparison of intubating laryngeal mask airway and Bullard laryngoscope for oro-tracheal intubation in adult patients with simulated limitation of cervical movements." BRITISH JOURNAL OF ANAESTHESIA 99(2): 292-296.

437.Nishikawa, K., et al. (2011). "Efficacy of the Airtraq(®) laryngoscope with a fiberoptic bronchoscope compared with that of Airtraq(®) alone for tracheal intubation: a manikin study." JOURNAL OF ANESTHESIA 25(1): 93‐97.

438.Noda, M., et al. (2013). "Successful management of intractable chylothorax in Gorham-Stout disease by awake thoracoscopic surgery." General thoracic and cardiovascular surgery 61(6): 356-358.

439.Nookala, A. and T. Welch (2018). "Trans-odontoid screw migration and airway management in the neurosurgical patient." JOURNAL OF NEUROSURGICAL ANESTHESIOLOGY 30(4): 464.

440.Noppens, R. R., et al. (2009). "[Airway management in a patient with immobilized cervical spine. Preclinical use of the McGrath video-laryngoscope]." Anaesthesist 58(5): 469-473.

441.Novotny, Z., et al. (2012). "Difficult airway in advanced bechterew's disease: Case report." ACTA CLINICA CROATICA 51(3): 463-466.

442.Ohno, S., et al. (2014). "A case of general anesthesia for a pregnant patient with PAPA syndrome." Japanese journal of anesthesiology 63(8): 921-923.

443.Ohno, S., et al. (2014). "[General anesthesia for a pregnant patient with PAPA syndrome]." Masui 63(8): 921-923.

444.Okpokpo, E. M., et al. (2021). "A rare case of cryptogenic organizing pneumonia in rheumatoid arthritis patient on golimumab therapy." American Journal of Respiratory and Critical Care Medicine 203(9).

445.Olomu, P., et al. (2020). "Morbid obesity: a protective factor in paediatric penetrating neck trauma?" Trends in Anaesthesia and Critical Care 30: e142.

446.Ong, J., et al. (2016). "Comparison between the Trachway video intubating stylet and Macintosh laryngoscope in four simulated difficult tracheal intubations: A manikin study." Tzu Chi Medical Journal 28(3): 109-112.

447.Orjuela, K., et al. (2014). "Right hemispheric acute ischemic stroke secondary to right common carotid artery free floating thrombus due to hypercoagulable state in an otherwise healthy 48-year-old woman." Neurology 82(10).

448.Osella, J., et al. (2023). "A RARE CASE OF ENDOBRONCHIAL LARGE B CELL LYMPHOMA." Chest 164(4): A4282-A4283.

449.Otsuka, Y., et al. (2011). "Distortion of the anterior airway anatomy and cervical spine motion during laryngoscopy with GlideScope® videolaryngoscope: A comparison of mid-size blade vs large blade." Japanese journal of anesthesiology 60(3): 361-366.

450.Otsuka, Y., et al. (2011). "[Distortion of the anterior airway anatomy and cervical spine motion during laryngoscopy with GlideScope videolaryngoscope: a comparison of mid-size blade vs large blade]." Masui 60(3): 361-366.

451.Ozgul, U., et al. (2019). "Comparison of videolaryngoscope-guided versus standard digital insertion techniques of the ProSeal™ laryngeal mask airway: a prospective randomized study." BMC ANESTHESIOLOGY 19(1): 244.

452.Ozkan, A. S. and S. Akbas (2018). "Nasotracheal Intubation in Children for Outpatient Dental Surgery: is Fiberoptic Bronchoscopy Useful?" Nigerian journal of clinical practice 21(2): 183‐188.

453.Özkan, D., et al. (2019). "Comparison of cervical spine motion during intubation with a C‑MAC D‑Blade® and an LMA Fastrach®." Anaesthesist 68(2): 90-96.

454.Ozkan, D., et al. (2021). "Intubating conditions with articulating vs. Intubating stylet during video laryngoscope intubation in anticipated difficult airway patients." The Kuwait medical journal 53(2): 184‐190.

455.Paik, H. and H. P. Park (2020). "Randomized crossover trial comparing cervical spine motion during tracheal intubation with a Macintosh laryngoscope versus a C-MAC D-blade videolaryngoscope in a simulated immobilized cervical spine." BMC ANESTHESIOLOGY 20(1).

456.Pariyadath, A., et al. (2015). "Tracheal perforation by an anterior cervical fixation device." Chest 148(4).

457.Park, J. W., et al. (2021). "Comparison of a New Video Intubation Stylet and McGrath® MAC Video Laryngoscope for Intubation in an Airway Manikin with Normal Airway and Cervical Spine Immobilization Scenarios by Novice Personnel: A Randomized Crossover Study." BioMed research international 2021.

458.Park, S. O., et al. (2013). "Efficacy of the Disposcope endoscope, a new video laryngoscope, for endotracheal intubation in patients with cervical spine immobilisation by semirigid neck collar: Comparison with the Macintosh laryngoscope using a simulation study on a manikin." EMERGENCY MEDICINE JOURNAL 30(4): 270-274.

459.Parnell, J. D. and J. Mills (2006). "Awake intubation using fast-track laryngeal mask airway as an alternative to fiberoptic bronchoscopy: A case report." AANA Journal 74(6): 429-431.

460.Passot, S., et al. (2002). "Target-controlled versus manually-controlled infusion of propofol for direct laryngoscopy and bronchoscopy." ANESTHESIA AND ANALGESIA 94(5): 1212‐1216, table of contents.

461.Patel, N. and D. Desai (2021). "Tracheal intubation with King Vision video laryngoscope in patients with cervical spine instability-Comparison of straight versus curved reinforced endotracheal tubes." Indian Journal of Anaesthesia 65(9): 650-655.

462.Patel, N. and D. J. Desai (2021). "Tracheal intubation with King Vision video laryngoscope in patients with cervical spine instability-Comparison of straight versus curved reinforced endotracheal tubes." Indian J Anaesth 65(9): 650-655.

463.Patrick, J. S., et al. (2022). "A novel assistant system for transoral surgery of the larynx." Laryngo- Rhino- Otologie 101: S349-S350.

464.Paul, A. L., et al. (2022). "Safety and Feasibility of very Early Bronchoscopy-assisted Percutaneous Dilatational Tracheostomy in Anterior Cervical Spine Fixation Patients." Indian Journal of Critical Care Medicine 26(10): 1086-1090.

465.Peck, M. J., et al. (2009). "Laryngoscopy and tracheal intubation using the McGrath laryngoscope in patients with cervical spine in-line immobilization." Canadian Journal of Anesthesia 56: S85.

466.Perungo, T., et al. (2014). "Surgical management of post intubation tracheoesophgeal fistulae." Diseases of the Esophagus 27: 35A.

467.Peters, J. E., et al. (2011). "Three cases of rheumatoid arthritis with laryngeal stridor." CLINICAL RHEUMATOLOGY 30(5): 723-727.

468.Petersen, H. and W. Lenz (1975). "Severe injury to the neck region by seat belt." Aktuelle Traumatologie 5(4): 271-279.

469.Phipps, S. J., et al. (2019). "The Role of ECMO in the "At-Risk" Tracheal Extubation: A Case Report." A and A Practice 12(2): 41-43.

470.Pickrell, B. B., et al. (2017). "Tracheal Cartilaginous Sleeve in Syndromic Craniosynostosis: An Underrecognized Source of Significant Morbidity and Mortality." The Journal of craniofacial surgery 28(3): 696-699

471.Piepho, T., et al. (2011). "Comparison of the McGrath® Series 5 and GlideScope® Ranger with the Macintosh laryngoscope by paramedics." Scandinavian journal of trauma, resuscitation and emergency medicine 19(1): 4.

472.Pillai, A. K., et al. (2019). "Comparison of haemodynamic responses to orotracheal intubation in anaesthetised and paralysed patients with simulated cervical spine injury: airtraq® video laryngoscope versus fibreoptic bronchoscope." Sri Lankan Journal of Anaesthesiology 27(2): 145‐150.

473.Pimentel, J. L. and A. Bugalho Almeida (2012). "Difficult intubation: 10 years of experience." American Journal of Respiratory and Critical Care Medicine 185.

474.Prasarn, M. L., et al. (2012). "Comparison of 4 airway devices on cervical spine alignment in a cadaver model with global ligamentous instability at C5-C6." SPINE 37(6): 476-481.

475.Preciado, D. (2014). "A randomized study of suprastomal stents in laryngotracheoplasty surgery for grade III subglottic stenosis in children." LARYNGOSCOPE 124(1): 207‐213.

476.Putnam, T., et al. (2020). "A RARE CASE OF CERVICAL HARDWARE MIGRATION CAUSING ESOPHAGEAL PERFORATION." Chest 158(4): A1922-A1923.

477.Qi, L., et al. (2016). "A comparison of effects of four different methods to locate tracheal tube." Zhonghua wei zhong bing ji jiu yi xue 28(9): 812‐816.

478.Radwan, T., et al. (2017). "Comparative study between dexmedetomidine, magnesium sulphate and fentanyl as sedatives throughout awake fiberoptic intubation for patients undergoing cervical spine surgeries Comparative study between dexmedetomidine, magnesium sulphate and fentanyl." Egyptian journal of anaesthesia 33(4): 345-349.

479.Raghavendra Babu, T., et al. (2019). "A comparative study of airtraq® and McCoy laryngoscopes for endotracheal intubation in adult patients with simulated difficult airway using a rigid cervical collar in elective surgeries under general anaesthesia." Sri Lankan Journal of Anaesthesiology 27(1): 28-34.

480.Rahman, M. (2016). "A case report of right lung agenesis with klippel-feil syndrome." Chest 149(4): A243.

481.Rajajee, V., et al. (2011). "Real-time ultrasound-guided percutaneous dilatational tracheostomy: A feasibility study." CRITICAL CARE 15(1).

482.Rajajee, V., et al. (2015). "Impact of real-time ultrasound guidance on complications of percutaneous dilatational tracheostomy: A propensity score analysis." CRITICAL CARE 19(1).

483.Rajaleelan, W., et al. (2022). "Emergency Airway Management in the Prone Position: A Mannequin- Based Randomized Cross Over Simulation Study." Canadian Journal of Anesthesia 69: S188-S189.

484.Rajan, S., et al. (2015). "Congenital and acquired cervical spine disorders in pediatric patients producing a difficult airway." JOURNAL OF NEUROSURGICAL ANESTHESIOLOGY 27(4): 437-438.

485.Raksakietisak, M., et al. (2018). "A retrospective study of airway related complications in cervical spine surgery." Journal of the Medical Association of Thailand 101(9): S19-S25.

486.Ramesh, K., et al. (2023). "Comparison of Tracheal Intubation Using King Vision (Non-channeled Blade) and Tuoren Video Laryngoscopes in Patients With Cervical Spine Immobilization by Manual In-Line Stabilization: A Randomized Clinical Trial." Cureus 15(8): e43471.

487.Randall, D. R., et al. (2017). "Altered pharyngeal structure and dynamics among patients with cervical kyphosis." LARYNGOSCOPE 127(8): 1832-1837.

488.Rathlev, N. K., et al. (2007). "Evaluation and Management of Neck Trauma." Emergency Medicine Clinics of North America 25(3): 679-694.

489.Reader, M. M., et al. (2012). "An evaluation of video laryngoscopy techniques compared with direct laryngoscopy in prehospital helicopter emergency medical personnel." Air medical journal 31(4): 170.

490.Redhu, S., et al. (2015). "AN AUDIT OF THE CURRENT PRACTICES OF AIRWAY MANAGEMENT IN PATIENTS UNDERGOING SURGERY FOR ATLANTO-AXIAL DISLOCATION IN A SINGLE INSTITUTION." Middle East journal of anaesthesiology 23(2): 163-170.

491.Reece, G. P. and C. H. Shatney (1988). "Blunt injuries of the cervical trachea: Review of 51 patients." SOUTHERN MEDICAL JOURNAL 81(12): 1542-1548.

492.Reinstrup, P., et al. (1996). "Perioperative airway management in patients undergoing surgery for rheumatoid arthritis of the cervical spine." Journal of Orthopaedic Rheumatology 9(2): 96-99.

493.Reyhan, N., et al. (2017). "Comparison of C-MAC, McGrath and Macintosh laryngoscope use in a standardized airway manikin with immobilized cervical spine by novice intubators." Am J Emerg Med 35(9): 1368-1370.

494.Rezak, A., et al. (2010). "Ultrasound-guided placement of percutaneous dilatational tracheostomy." Journal of Surgical Research 158(2): 416-417.

495.Riou, B., et al. (1991). "Endotracheal intubation using a fiberoptic laryngoscope." Annales francaises d'anesthesie et de reanimation 10(3): 308-310.

496.Riou, B., et al. (1991). "Intratracheal intubation using a fiberoptic laryngoscope." Annales francaises d'anesthesie et de reanimation 10(3): 308-310.

497.Riou, B., et al. (1991). "Fiberoptic laryngoscopic intubation (Bullard®) in patients with cervical spine trauma." Annales francaises d'anesthesie et de reanimation 10(SUPPL.): R192.

498.Robitaille, A. (2011). "Airway management in the patient with potential cervical spine instability: Continuing Professional Development." Canadian Journal of Anesthesia 58(12): 1125-1139.

499.Romito, J., et al. (2018). "Comparison of four endotracheal intubation devices on cervical spine movement in a cadaver model." Critical care medicine 46: 769.

500.Romito, J. W., et al. (2020). "Cervical Spine Movement in a Cadaveric Model of Severe Spinal Instability: A Study Comparing Tracheal Intubation with 4 Different Laryngoscopes." JOURNAL OF NEUROSURGICAL ANESTHESIOLOGY 32(1): 57-62.

501.Rossi, M., et al. (1997). "The use of fiberoptic bronchoscopy during percutaneous dilatational tracheostomy with laryngeal mask." Diagn Ther Endosc 4(1): 13-18.

502.Royds, J., et al. (2019). "Achieving proficiency in rigid bronchoscopyâ€”a study in manikins." Irish journal of medical science 188(3): 979‐986.

503.Roye Jr, W. P., et al. (1988). "Cervical spinal cord injury - A public catastrophe." Journal of trauma 28(8): 1260-1264.

504.Roye, W. P., Jr., et al. (1988). "Cervical spinal cord injury--a public catastrophe." J Trauma 28(8): 1260-1264.

505.Rudolph, C., et al. (2005). "Movement of the upper cervical spine during laryngoscopy: A comparison of the Bonfils intubation fibrescope and the Macintosh laryngoscope." Anaesthesia 60(7): 668-672.

506.Rudolph, C., et al. (2005). "Movement of the upper cervical spine during laryngoscopy: a comparison of the Bonfils intubation fibrescope and the Macintosh laryngoscope." Anaesthesia 60(7): 668-672.

507.Ruetzler, K., et al. (2015). "Comparison of five video laryngoscopes and conventional direct laryngoscopy. Investigations on simple and simulated difficult airways on the intubation trainer." Anaesthesist 64(7): 513-519.

508.Russell, T., et al. (2011). "A comparison of the forces applied to a manikin during laryngoscopy with the GlideScope and Macintosh laryngoscopes." Anaesth Intensive Care 39(6): 1098-1102.

509.Russell, T., et al. (2011). "A comparison of the forces applied to a manikin during laryngoscopy with the GlideScope® and Macintosh laryngoscopes." ANAESTHESIA AND INTENSIVE CARE 39(6): 1098-1102.

510.Ryu, T., et al. (2019). "Comparing the placement of a left-sided double-lumen tube via fiberoptic bronchoscopy guidance versus conventional intubation using a Macintosh laryngoscope, to reduce the incidence of malpositioning: study protocol for a randomized controlled pilot trial." Trials 20(1): 51.

511.Saha, A. K., et al. (1998). "Comparison of awake endotracheal intubation in patients with cervical spine disease: The lighted intubating stylet versus the fiberoptic bronchoscope." ANESTHESIA AND ANALGESIA 87(2): 477-479.

512.Şahin, T., et al. (2018). "Fluoroscopic Comparison of Cervical Spine Motion Using LMA CTrach, C-MAC Videolaryngoscope and Macintosh Laryngoscope." Turk J Anaesthesiol Reanim 46(1): 44-50.

513.Saini, S., et al. (2008). "Left molar approach improves laryngeal view in patients with simulated limitation of cervical movements." ACTA ANAESTHESIOLOGICA SCANDINAVICA 52(6): 829-833.

514.Saito, T., et al. (2012). "Efficacy of Coopdech videolaryngoscope: Comparisons with a Macintosh laryngoscope and the Airway Scope in a manikin with difficult airways." JOURNAL OF ANESTHESIA 26(4): 617-620.

515.Saito, T., et al. (2010). "Comparison of airway scope and videolaryngoscope portable VLP-100 in the presence of a neck collar - A manikin study." Japanese journal of anesthesiology 59(12): 1544-1547.

516.Saito, T., et al. (2010). "[Comparison of airway scope and videolaryngoscope portable VLP-100 in the presence of a neck collar--a manikin study]." Masui 59(12): 1544-1547.

517.Saito, T. and Y. Okuda (2010). "Comparison of airwayscope® and videolaryngoscope portable VLP100® in the presence of a neck collar-a manikin study." ANESTHESIA AND ANALGESIA 110(3): S124.

518.Saleh, M. and C. Jenkins (2019). "The paradox." Rheumatology Advances in Practice 3: i31-i32.

519.Sanghvi, R. and J. L. Benumof (1999). "The Bullard laryngoscope: A useful solution to the problem of the difficult tracheal intubation." Progress in Anesthesiology 13(19): 347-354+356+355.

520.Santamaria, L. B., et al. (2007). "Preanesthetic evaluation and assessment of children with Down's syndrome." TheScientificWorldJournal 7: 242-251.

521.Santoni, B. G., et al. (2009). "Manual in-line stabilization increases pressures applied by the laryngoscope blade during direct laryngoscopy and orotracheal intubation." Anesthesiology 110(1): 24-31.

522.Saoulidou, E., et al. (2021). "Case report: Awake nasal intubation with the use of video-laryngoscope, in a patient with a background of ankylosing spondylitis, presenting for inguinal hernia repair." ANESTHESIA AND ANALGESIA 133(3 SUPPL 2): 464.

523.Saracoglu, K. T., et al. (2014). "The use of Airtraq laryngoscope versus Macintosh laryngoscope and fiberoptic bronchoscope by experienced anesthesiologists." Middle East journal of anaesthesiology 22(5): 503‐509.

524.Sarangi, S., et al. (2016). "An unusual presentation of autonomic dysreflexia in a patient with cold abscess of cervical spine for anterolateral decompression." Indian Journal of Anaesthesia 60(12): 955-957.

525.Saurav, et al. (2019). "Anaesthetic management of bilateral temporomandibular joint ankylosis with cervical spine fusion for total alloplastic joint replacement in a patient with ankylosing spondylitis." Indian Journal of Anaesthesia 63(2): 149-150.

526.Sawin, P. D., et al. (1996). "Cervical spine motion with direct laryngoscopy and orotracheal intubation: An in vivo cinefluoroscopic study of subjects without cervical abnormality." Anesthesiology 85(1): 26-36.

527.Schild, L. R., et al. (2020). "Transoral videolaryngoscopic surgery of the larynx: Prototype with flexible surgical instruments provides appropriate visualization and accessibility." Laryngo- Rhino- Otologie 99(SUPPL 2): S129-S130.

528.Schild, L. R., et al. (2021). "Prototype with flexible surgical instruments provides appropriate visualization and accessibility." Laryngo- Rhino- Otologie 100(SUPPL 2): S31-S32.

529.Schober, P., et al. (2009). "Inverse intubation of entrapped trauma casualties - A comparison of direct laryngoscopy, indirect optical laryngoscopy and video laryngoscopy in a simulated scenario." EUROPEAN JOURNAL OF ANAESTHESIOLOGY 26: 222.

530.Schoettker, P., et al. (2012). "Comparison of the single-use Ambu aScope2® versus the fiberoptic bronchoscope for tracheal intubation in patients with cervical spine immobilisation by a semi-rigid collar." EUROPEAN JOURNAL OF ANAESTHESIOLOGY 29: 228-229.

531.Schuepbach, R., et al. (2015). "Intubation with VivaSight or conventional left-sided double-lumen tubes: a randomized trial." Canadian Journal of Anesthesia 62(7): 762‐769.

532.Schuler, P. J., et al. (2021). "Transoral video-laryngoscopic surgery of the larynx." Biomedizinische Technik 66(SUPPL 1): S413.

533.Schuschnig, C., et al. (1999). "Intubating laryngeal mask and rapid sequence induction in patients with cervical spine injury." Anaesthesia 54(8): 793-797.

534.Sen, R., et al. (2020). "Comparison of TruView and King Vision video laryngoscopes in subaxial cervical spine injury: A randomized controlled trial." Surg Neurol Int 11: 375.

535.Sener, E. B., et al. (2002). "Awake tracheal intubation through the intubating laryngeal mask airway in a patient with halo traction." Canadian Journal of Anesthesia 49(6): 610-613.

536.Seo, K. H., et al. (2020). "Comparison of C-MAC D-blade videolaryngoscope and McCoy laryngoscope efficacy for nasotracheal intubation in simulated cervical spinal injury: A prospective randomized comparative study." BMC ANESTHESIOLOGY 20(1).

537.Serdiuk, A. A. and V. Bosek (2012). "An adult patient with Klippel-Feil syndrome presenting for repeat operation: a cautionary tale of the GlideScope." JOURNAL OF CLINICAL ANESTHESIA 24(3): 238-241.

538.Shah, M. B., et al. (2008). "Flexible bronchoscopy and interdisciplinary collaboration in pediatric large airway disease." INTERNATIONAL JOURNAL OF PEDIATRIC OTORHINOLARYNGOLOGY 72(12): 1771-1776.

539.hallik, N., et al. (2016). "Is it time for routine use of the retromolar fiberscope?" Saudi journal of anaesthesia 10(2): 213-217.

540.Sharma, N., et al. (2023). "A COMPARISON OF KETAMINE-DEXMEDETOMIDINE AND KETAMINE-PROPOFOL COMBINATIONS FOR AWAKE FIBEROPTIC INTUBATION IN ADULT PATIENTS WITH SIMULATED CERVICAL INJURY." International Journal of Academic Medicine and Pharmacy 5(3): 628-632.

541.Sheshadri, V., et al. (2017). "Airway adverse events following posterior occipito-cervical spinal fusion." JOURNAL OF CLINICAL NEUROSCIENCE 39: 124-129.

542.Shesterina, M. V., et al. (1976). "Bronchofibroscopy in patients with tuberculosis and other pulmonary affections (procedures, indications, contraindications) (Russian)." Problemy tuberkuleza 54(11): 37-40.

543.Shih, T. L., et al. (2022). "The Use of the Shikani Video-Assisted Intubating Stylet Technique in Patients with Restricted Neck Mobility." HEALTHCARE 10(9).

544.Shih, T.-L., et al. (2022). "The Use of the Shikani Video-Assisted Intubating Stylet Technique in Patients with Restricted Neck Mobility." Healthcare (Basel, Switzerland) 10(9).

545.Shikani, A. H., et al. (2023). "Linear versus Turbulent Airflow Tracheostomy Heat and Moisture Exchangers: a Crossover Study." LARYNGOSCOPE.

546.Shippey, B., et al. (2013). "A comparison of the McGrath® videolaryngoscope and the Macintosh laryngoscope in patients with cervical spine immobilisation." Anaesthesia 68(8): 883.

547.Shirgoska, B. and J. Netkovski (2012). "New techniques and devices for difficult airway management." ACTA CLINICA CROATICA 51(3): 457-461.

548.Shivanand, L. K., et al. (2022). "Difficult Airway Management-A Challenge to Anaesthesiologists." Journal of Clinical and Diagnostic Research 16(7): UR01-UR05.

549.Shravanalakshmi, D., et al. (2017). "Comparison of intubation success and glottic visualization using King Vision and C-MAC videolaryngoscopes in patients with cervical spine injuries with cervical immobilization: A randomized clinical trial." Surgical Neurology International 8(1).

550.Shravanalakshmi, D., et al. (2017). "Comparison of intubation success and glottic visualization using King Vision and C-MAC videolaryngoscopes in patients with cervical spine injuries with cervical immobilization: A randomized clinical trial." Surg Neurol Int 8: 19.

551.Shulman, G. B. and N. R. Connelly (2001). "A comparison of the Bullard laryngoscope versus the flexible fiberoptic bronchoscope during intubation in patients afforded inline stabilization." JOURNAL OF CLINICAL ANESTHESIA 13(3): 182-185.

552.Shulwan, G. B. and N. R. Connelly (2001). "A comparison of the Bullard laryngoscope versus the flexible fiberoptic bronchoscope during intubation in patients afforded Inline stabilization." JOURNAL OF CLINICAL ANESTHESIA 13(3): 182-185.

553.Sinclair, C. F., et al. (2017). "Contralateral R1 and R2 components of the laryngeal adductor reflex in humans under general anesthesia." LARYNGOSCOPE 127(12): E443-E448.

554.Singh, J., et al. (2018). "Awake fiberoptic intubation in cervical spine injury: A comparison between atomized local anesthesia versus airway nerve blocks." Kathmandu University Medical Journal 16(64): 323-327.

555.Singh, R., et al. (2010). "Haemodynamic response to nasotracheal intubation under general anaesthesia - a comparison between fiberoptic bronchoscopy and direct laryngoscopy." Journal of anaesthesiology, clinical pharmacology 26(3): 335‐339.

556.Singh, S., et al. (2023). "Comparison between C-MAC D-blade video laryngoscope and McCoy laryngoscope for nasotracheal intubation in traumatic cervical spine surgery - A randomised controlled trial." Indian J Anaesth 67(9): 821-824.

557.Sinha, R., et al. (2019). "Comparison of the C-MAC video laryngoscope size 2 Macintosh blade with size 2 C-MAC D-Blade for laryngoscopy and endotracheal intubation in children with simulated cervical spine injury: A prospective randomized crossover study." Journal of Anaesthesiology Clinical Pharmacology 35(4): 509-514.

558.Sinha, R., et al. (2019). "Comparison of the C-MAC video laryngoscope size 2 Macintosh blade with size 2 C-MAC D-Blade for laryngoscopy and endotracheal intubation in children with simulated cervical spine injury: A prospective randomized crossover study." J Anaesthesiol Clin Pharmacol 35(4): 509-514.

559.Smereka, J., et al. (2017). "C-MAC compared with direct laryngoscopy for intubation in patients with cervical spine immobilization: A manikin trial." AMERICAN JOURNAL OF EMERGENCY MEDICINE 35(8): 1142-1146.

560.Smith, C. E. and S. J. DeJoy (2001). "New equipment and techniques for airway management in trauma." Current opinion in anaesthesiology 14(2): 197-209.

561.Smith, C. E., et al. (1999). "Evaluation of tracheal intubation difficulty in patients with cervical spine immobilization - Fiberoptic (WuScope) versus conventional laryngoscopy." Anesthesiology 91(5): 1253-1259.

562.Smith, C. E., et al. (1999). "Evaluation of tracheal intubation difficulty in patients with cervical spine immobilization: Fiberoptic (WuScope) versus conventional laryngoscopy." Anesthesiology 91(5): 1253-1259.

563.Sobiech, S., et al. (2010). "Tracheal laceration associated with cervical spine injury-case report." Ortopedia, traumatologia, rehabilitacja 12(2): 166-174.

564.Souvatzis, X. and H. Askitopoulou (2008). "Airway management in cervical spinal cord injured patients: A survey of European emergency physicians' clinical practice." EUROPEAN JOURNAL OF EMERGENCY MEDICINE 15(6): 344-347.

565.Sreenath, S. B., et al. (2023). "Free Tissue Transfer for Skull Base Osteoradionecrosis: A Novel Approach in the Endoscopic Era." LARYNGOSCOPE 133(3): 562-568.

566.Sriramka, B. and S. K. Pattnaik (2017). "Retrograde intubation in a patient with neglected cervical spine injury for sacral advancement flap." Sri Lankan Journal of Anaesthesiology 25(2): 115-117.

567.Starr, L. J., et al. (2023). "Siblings with profound connective tissue disease: First report of biallelic TGFBR1-related Loeys-Dietz syndrome." American Journal of Medical Genetics, Part A 191(3): 786-793.

568.Stegmann, G., et al. (2021). "Global airway management of the unstable cervical spine survey (GAUSS)." Southern African Journal of Anaesthesia and Analgesia 27(6): 278-285.

569.Stephens, C. T., et al. (2018). "Use of the Bougie Endotracheal Tube Introducer in Unstable Cervical Spine Airway Management?" ANESTHESIA AND ANALGESIA 127(6): E112.

Struck, M. F., et al. (2011). "Prehospital Glidescope video laryngoscopy for difficult airway management in a helicopter rescue program with anaesthetists." EUROPEAN JOURNAL OF EMERGENCY MEDICINE 18(5): 282-284.

570.Subedi, A., et al. (2014). "Successful intubation with McCoy laryngoscope in a patient with ankylosing spondylitis." Journal of Nepal Health Research Council 12(26): 70-72.

571.Suppan, L., et al. (2016). "Alternative intubation techniques vs Macintosh laryngoscopy in patients with cervical spine immobilization: Systematic review and meta-analysis of randomized controlled trials." BRITISH JOURNAL OF ANAESTHESIA 116(1): 27-36.

572.Swain, A., et al. (2015). "A cinefluoroscopic assessment of cervical spine motion during orotracheal intubation: ILMA guided flexible bronchoscopic intubation versus video laryngoscopy." JOURNAL OF NEUROSURGICAL ANESTHESIOLOGY 27(4): 450-451.

573.Swain, A., et al. (2020). "Intubating Laryngeal Mask Airway-assisted Flexible Bronchoscopic Intubation Is Associated With Reduced Cervical Spine Motion When Compared With C-MAC Video Laryngoscopy-guided Intubation: a Prospective Randomized Cross Over Trial." JOURNAL OF NEUROSURGICAL ANESTHESIOLOGY 32(3): 242‐248.

574.Szarpak, L. (2018). "Laryngoscopes for difficult airway scenarios: a comparison of the available devices." EXPERT REVIEW OF MEDICAL DEVICES 15(9): 631-643.

575.Taguchi, A., et al. (2015). "Comparison of seven intubation devices in difficult airway model." Japanese journal of anesthesiology 64(4): 352-356.

576.Takahashi, K., et al. (2010). "Comparison of the airway scope and macintosh laryngoscope with in-line cervical stabilization by the semisolid neck collar: Manikin study." Journal of Trauma - Injury, Infection and Critical Care 68(2): 363-366.

577.Takenaka, I., et al. (2009). "Approach combining the airway scope and the bougie for minimizing movement of the cervical spine during endotracheal intubation." Anesthesiology 110(6): 1335‐1340.

578.Tatekawa, Y., et al. (2008). "A new technique for treatment of tracheal compression by the innominate artery: external reinforcement with autologous cartilage graft and muscle flap suspension." PEDIATRIC SURGERY INTERNATIONAL 24(4): 431-435.

579.Tawfeek, M. M. and A. M. Abdelbaky (2011). "Is fiberoptic bronchoscope a good intubating choice in anesthetized patients with anticipated difficult intubation?" Egyptian journal of anaesthesia 27(3): 157‐161.

580.Thiboutot, F., et al. (2009). "Effect of manual in-line stabilization of the cervical spine in adults on the rate of difficult orotracheal intubation by direct laryngoscopy: A randomized controlled trial." Canadian Journal of Anesthesia 56(6): 412-418.

581.Thierbach, A. R. and C. Werner (2005). "Infraglottic airway devices and techniques." Best Practice and Research: Clinical Anaesthesiology 19(4): 595-609.

582.Tiwari, A. and T. Gravelyn (2011). "Cough, dyspnea and interstitial lung disease in a 20year-oldsmoker." Journal of general internal medicine 26: S365.

583.Tiwari, A. and T. Gravelyn (2011). "Cough, dyspnea, and interstitial lung disease in a 20-year-old smoker." Journal of Hospital Medicine 6(4): S277.

584.Tolon, M. A., et al. (2012). "Comparative study between the use of Macintosh Laryngoscope and Airtraq in patients with cervical spine immobilization." Alexandria journal of medicine 48(2): 179-185.

585.Tonks, K. E., et al. (2023). "Perioperative management of a parturient with VACTERL association for a caesarean section." Anaesthesia Reports 11(1).

586.Trippick, S. and M. Duffy (2012). "A simulator-based trial to compare intubation attempts between direct laryngoscopy and three video laryngoscopes in a variety of airway settings." Academic Emergency Medicine 19(6): 753.

587.Truszewski, Z., et al. (2016). "Comparison of two techniques to intubate a child under simulated cervical spine injury. The randomized, crossover study." Pediatria polska 91(3): 233-239.

588.Truszewski, Z., et al. (2016). "Comparison of the three types of laryngoscopes during child intubation under simulated cervical spine injury." Pediatria polska 91(1): 40-45.

589.Tsugawa, C., et al. (2004). "Transection of the innominate artery for tracheomalacia caused by persistent opisthotonus." PEDIATRIC SURGERY INTERNATIONAL 20(1): 55-57.

590.Tsui, B., et al. (2019). "Transnasal humidified rapid-insufflation ventilatory exchange improves oxygenation in pediatric bronchoscopy." Canadian Journal of Anesthesia 66(2): S169‐S171.

591.Tsukamoto, M., et al. (2015). "Anesthetic management of a combative patient with down syndrome who did not undergo a preoperative examination." Journal of Japanese Dental Society of Anesthesiology 43(1): 60-62.

592.Tumu, D. P., et al. (2021). "Cervical spine motion: A fluoroscopic comparison between Airtraq laryngoscope with fiberoptic bronchoscope and Macintosh laryngoscope-a cross over randomized controlled study." ANESTHESIA AND ANALGESIA 133(3 SUPPL 2): 477-479.

593.Turezyn, K., et al. (2018). "Successful airway management of patients with morquio syndrome: Are there useful predictors?" ANESTHESIA AND ANALGESIA 126(4): 17-18.

594.Turkstra, T. P., et al. (2005). "Cervical spine motion: A fluoroscopic comparison during intubation with lighted Stylet, GlideScope, and Macintosh laryngoscope." ANESTHESIA AND ANALGESIA 101(3): 910-915.

595.Turkstra, T. P., et al. (2009). "Cervical spine motion: A fluoroscopic comparison of the airtraq laryngoscope versus the macintosh laryngoscope." Anesthesiology 111(1): 97-101.

596.Turkstra, T. P., et al. (2009). "Comparison of AirTraq® laryngoscope to macintosh laryngoscope for intubation of patients with potential cervical spine injury: A fluoroscopic randomized controlled trial." Canadian Journal of Anesthesia 56: S112.

597.Turkstra, T. P., et al. (2009). "<i>Cervical Spine Motion A Fluoroscopic Comparison of the AirTraq Laryngoscope</i> versus <i>the Macintosh Laryngoscope</i>." Anesthesiology 111(1): 97-101.

598.Turkstra, T. P., et al. (2007). "Cervical spine motion: a fluoroscopic comparison of Shikani Optical Stylet vs Macintosh laryngoscope." Can J Anaesth 54(6): 441-447.

599.Turnbull, D. and A. Vinogradov (2013). "Intubation after cervical spine surgery: A retrospective review." JOURNAL OF NEUROSURGICAL ANESTHESIOLOGY 25(4): 467.

600.Turnbull, D. and A. Vinogradov (2013). "Intubation difficulty and cervical spine fusion: A retrospective review." BRITISH JOURNAL OF ANAESTHESIA 110(5): 884p.

601.Turner, C. R., et al. (2009). "Motion of a cadaver model of cervical injury during endotracheal intubation with a Bullard laryngoscope or a Macintosh blade with and without in-line stabilization." Journal of Trauma - Injury, Infection and Critical Care 67(1): 61-66.

602.Turner, C. R., et al. (2009). "Motion of a Cadaver Model of Cervical Injury During Endotracheal Intubation With a Bullard Laryngoscope or a Macintosh Blade With and Without In-line Stabilization." JOURNAL OF TRAUMA-INJURY INFECTION AND CRITICAL CARE 67(1): 61-66.

603.Vadi, M. G., et al. (2017). "Comparison of the GlideScope Cobalt® and Storz DCI® Video Laryngoscopes in Children Younger Than 2 Years of Age During Manual In-Line Stabilization <i>A Randomized Trainee Evaluation Study</i>." PEDIATRIC EMERGENCY CARE 33(7): 467-473.

604.Vaisbuch, Y., et al. (2019). "Ergonomic hazards in otolaryngology." LARYNGOSCOPE 129(2): 370-376.

605.Van Elstraete, A. C., et al. (1998). "Nasotracheal intubation in patients with immobilized cervical spine: A comparison of tracheal tube cuff inflation and fiberoptic bronchoscopy." ANESTHESIA AND ANALGESIA 87(2): 400-402.

606.Vaughan, C. W. (1993). "Vocal fold exposure in phonosurgery." J Voice 7(2): 189-194.

607.Vengathajalam, S., et al. (2016). "Delayed post trauma retropharyngeal hematoma with acute airway obstruction in a non cervical-spine injury." Rawal Medical Journal 41(2): 253-255.

608.Verhofste, B. P., et al. (2019). "Perioperative acute neurological deficits in instrumented pediatric cervical spine fusions." Journal of Neurosurgery: Pediatrics 24(5): 528-538.

609.Vijayakumar, V., et al. (2016). "A comparison of macintosh and airtraq laryngoscopes for endotracheal intubation in adult patients with cervical spine immobilization using manual in line axial stabilization: A prospective randomized study." JOURNAL OF NEUROSURGICAL ANESTHESIOLOGY 28(4): 296-302.

610.Vivek, B., et al. (2017). "Comparison of success of tracheal intubation using Macintosh laryngoscope-assisted Bonfils fiberscope and Truview video laryngoscope in simulated difficult airway." Journal of Anaesthesiology Clinical Pharmacology 33(1): 107-111.

611.Vlatten, A., et al. (2012). "A comparison of the GlideScope video laryngoscope and standard direct laryngoscopy in children with immobilized cervical spine." PEDIATRIC EMERGENCY CARE 28(12): 1317-1320.

612.von Allmen, D. C., et al. (2021). "Factors associated with success following transcervical innominate artery suspension." INTERNATIONAL JOURNAL OF PEDIATRIC OTORHINOLARYNGOLOGY 150.

613.Votruba, J., et al. (2020). "Video Laryngoscopic Intubation Using the King Vision(TM) Laryngoscope in a Simulated Cervical Spine Trauma: A Comparison Between Non-Channeled and Channeled Disposable Blades." Diagnostics (Basel) 10(3).

614.Wackett, A., et al. (2005). "Bullard laryngoscopy by naïve operators in the cervical spine immobilized patient." JOURNAL OF EMERGENCY MEDICINE 29(3): 253-257.

615.Wahba, S. S., et al. (2012). "Comparative study of awake endotracheal intubation with Glidescope video laryngoscope versus flexible fiber optic bronchoscope in patients with traumatic cervical spine injury." Egyptian journal of anaesthesia 28(4): 257-260.

616.Wahlen, B. M. and E. Gercek (2004). "Three-dimensional cervical spine movement during intubation using the Macintosh and Bullard laryngoscopes, the bonfils fibrescope and the intubating laryngeal mask airway." Eur J Anaesthesiol 21(11): 907-913.

617.Wahlen, B. M. and E. Gercek (2004). "Three-dimensional cervical spine movement during intubation using the Macintosh and Bullard<SUP>TM</SUP> laryngoscopes, the bonfils fibrescope and the intubating laryngeal mask airway." EUROPEAN JOURNAL OF ANAESTHESIOLOGY 21(11): 907-913.

618.Wahlen, B. M. and E. Gercek (2004). "Three-dimensional cervical spine movement during intubation using the Macintosh and Bullard™ laryngoscopes, the Bonfils fibrescope and the Intubating Laryngeal Mask Airway." EUROPEAN JOURNAL OF ANAESTHESIOLOGY 21(11): 907-913.

619.Wang, H., et al. (2020). "Lightwand vs. Glidescope: Tracheal intubation in cadavers with a cervical spine collar." Trends in Anaesthesia and Critical Care 30: e115-e116.

620.Wang, P. K., et al. (2013). "Comparison of 3 video laryngoscopes with the Macintosh in a manikin with easy and difficult simulated airways." AMERICAN JOURNAL OF EMERGENCY MEDICINE 31(2): 330-338.

621.Wang, W., et al. (2020). "Main branch of ACN-to-RLN for management of laryngospasm due to unilateral vocal cord paralysis." LARYNGOSCOPE 130(10): 2412-2419.

622.Wang, W., et al. (2020). "Main branch of ACN-to-RLN for management of laryngospasm due to unilateral vocal cord paralysis." LARYNGOSCOPE 130(10): 2412-2419.

623.Wang, X., et al. (2020). "Comparison of two kinds of airway surface anesthesia by fiberoptic bronchoscopy for awake trachea intubation." Trends in Anaesthesia and Critical Care 30: e74.

624.Warnick, B. M., et al. (2012). "A pilot study to validate a new technique for measuring head movement during tracheal intubation." Canadian Journal of Anesthesia 59.

625.Wasem, S., et al. (2013). "Comparison of the Airtraq and the Macintosh laryngoscope for double-lumen tube intubation: a randomised clinical trial." EUROPEAN JOURNAL OF ANAESTHESIOLOGY 30(4): 180‐186.

626.Watts, A. D., et al. (1997). "Comparison of the Bullard and Macintosh laryngoscopes for endotracheal intubation of patients with a potential cervical spine injury." Anesthesiology 87(6): 1335-1342.

627.Watts, A. D. J., et al. (1997). "Comparison of the Bullard and Macintosh laryngoscopes for endotracheal intubation of patients with a potential cervical spine injury." Anesthesiology 87(6): 1335-1342.

628.Weale, A. E. and C. H. Marsh (1996). "Cervical osteotomy for the flexed cervical spine in ankylosing spondylitis." Journal of Orthopaedic Rheumatology 9(2): 91-95.

629.Wei, M. and N. Kamangar (2018). "New spinal kaposi sarcoma lesions despite improvement of endobronchial kaposi sarcoma after treatment with chemotherapy." American Journal of Respiratory and Critical Care Medicine 197(MeetingAbstracts).

630.Wendling, A. L., et al. (2013). "A comparison of 4 airway devices on cervical spine alignment in cadaver models of global ligamentous instability at C1-2." ANESTHESIA AND ANALGESIA 117(1): 126-132.

631.Wetsch, W. A., et al. (2012). "Comparison of different video laryngoscopes for emergency intubation in a standardized airway manikin with immobilized cervical spine by experienced anaesthetists. A randomized, controlled crossover trial." RESUSCITATION 83(6): 740-745.

632.White, A. N. J., et al. (2015). "Cervical spine overflexion in a halo orthosis contributes to complete upper airway obstruction during awake bronchoscopic intubation: a case report." Canadian Journal of Anesthesia 62(3): 289-293.

633.Wimalaratne, T., et al. (2015). "Off the handle' technique for a feil intubation scenario." Anaesthesia 70: 99.

634.Woischneck, D., et al. (2013). "Severe tracheo-oesophageal compression by combination of cervical spondylosis and struma nodosa." EUROPEAN SPINE JOURNAL 22(11): 2616.

635.Wong, D. M., et al. (2009). "Cervical spine motion during flexible bronchoscopy compared with the Lo-Pro GlideScope." BRITISH JOURNAL OF ANAESTHESIA 102(3): 424‐430.

636.Wong, D. M., et al. (2009). "Cervical spine motion during flexible bronchoscopy compared with the Lo-Pro GlideScope®." BRITISH JOURNAL OF ANAESTHESIA 102(3): 424-430.

637.Wright, T. M. and K. Vinayakom (1995). "Endotracheal tube replacement in patients with cervical spine injury [9]." Anesthesiology 82(5): 1307-1308.

638.Wu, B. G., et al. (2023). "Styletubation in Bariatric Surgery: A Case Report." HEALTHCARE 11(16).

639. Bochart RM, Busman-Sahay K, Bondoc S, Morrow DW,. Mitigation of endemic GI-tract pathogen-mediated inflammation through development of multimodal treatment regimen and its impact on SIV acquisition in rhesus macaques. PLoS Pathog. 2021 May 10;17(5):e1009565. .

640. Woods KS, Defarges AM, Abrams-Ogg AC, Dobson H, Brisson BA, Viel L, Bienzle D. Comparison of bronchoalveolar lavage fluid obtained by manual aspiration with a handheld syringe with that obtained by automated suction pump aspiration from healthy dogs. Am J Vet Res. 2014 Jan;75(1):85-90. .

641. Moats CR, Randall KT, Swanson TM, Crank HB, Armantrout KM, Barber-Axthelm AM, Burnett ND, Hobbs TR, Martin LD, Gilbride RM, Hansen S, Smedley JV. Non-bronchoscopic Bronchoalveolar Lavage as a Refinement for Safely Obtaining High-quality Samples from Macaques. Comp Med. 2020 Dec 1;70(6):520-525. .

642. Hooi KS, Defarges AM, Sanchez AL, Nykamp SG, Weese JS, Abrams-Ogg ACG, Bienzle D. Comparison of bronchoscopic and nonbronchoscopic bronchoalveolar lavage in healthy cats. Am J Vet Res. 2018 Nov;79(11):1209-1216. .
