# Supplementary material for: Comparison of outcomes between video laryngoscopy and flexible fiberoptic bronchoscopy for endotracheal intubation in adults with cervical neck immobilization: A systematic review and meta-analysis of randomized controlled trials
Source: PLoS One. 2024 Nov 15;19(11):e0313280. doi: 10.1371/journal.pone.0313280 (PMC11567517; doi:10.1371/journal.pone.0313280)
Supplement: Attachment — Submitted filename: Response to Reviewers.docx [file pone.0313280.s005.docx]

Responses to the comments of Reviewer #1

*Thank you for taking the time to read and comment on our manuscript.*

1. Reviewer #1: The decision to address a topic that has not been previously subjected to meta-analysis has proven to be a fruitful one. Nevertheless, when comparing endotracheal intubation methods, it is essential to consider patient-related factors, if present in the included study, as they may influence the efficacy of the procedure. In the absence of patient-related factors in the included studies, it is essential to include this information in the meta-analysis. The evaluation of the effectiveness of the methods being compared will be enhanced by the inclusion of patient-related factors.

*Thank you for your comments and suggestions. When comparing endotracheal intubation methods, the presence of patient-related factors in the included studies may influence the outcomes of the procedure. In our meta-analysis, we did not incorporate these patient-related factors, which represents a limitation of this study. In future research, we aim to improve the evaluation of the effectiveness of the compared methods by including patient-related factors. This presents a new direction for subsequent investigations.*

Responses to the comments of Reviewer #2

*Thank you for your recognition of our paper.*

2. Reviewer #2 I consider this manuscript technically sound, and the data supports conclusions. I believe the statistical analysis was performed appropriately and rigorously.I believe the manuscript has some minor grammar and writing errors. I consider this study worth publishing and will make an important merit to the literature.

*Thank you for your valuable comments. For grammatical and writing errors in the manuscript, we have asked professional English editors from AJE to correct them to avoid errors in language, grammar, punctuation and spelling. The verification code for editing the certificate is: F2F6-4F68-83FD-BFA1-D650.*
